# The potential impact of novel tuberculosis vaccines on health equity and financial protection in low-income and middle-income countries

**DOI:** 10.1136/bmjgh-2023-012466

**Published:** 2023-07-12

**Authors:** Allison Portnoy, Rebecca A Clark, Chathika K Weerasuriya, Christinah Mukandavire, Matthew Quaife, Roel Bakker, Inés Garcia Baena, Nebiat Gebreselassie, Matteo Zignol, Mark Jit, Richard G White, Nicolas A Menzies

**Affiliations:** 1 Department of Global Health, Boston University School of Public Health, Boston, MA, USA; 2 Center for Health Decision Science, Harvard T.H. Chan School of Public Health, Boston, MA, USA; 3 Department of Infectious Disease Epidemiology, London School of Hygiene & Tropical Medicine, London, UK; 4 TB Modelling Group, London School of Hygiene & Tropical Medicine, London, UK; 5 Centre for the Mathematical Modelling of Infectious Diseases, London School of Hygiene & Tropica Medicine, London, UK; 6 Coalition for Epidemic Preparedness Innovations, London, UK; 7 KNCV Tuberculosis Foundation, Den Haag, The Netherlands; 8 Global Tuberculosis Programme, World Health Organization, Geneva, Switzerland; 9 School of Public Health, The University of Hong Kong, Hong Kong SAR, People's Republic of China; 10 Department of Global Health and Population, Harvard T.H. Chan School of Public Health, Boston, MA, USA

**Keywords:** tuberculosis, vaccines, health economics

## Abstract

**Introduction:**

One in two patients developing tuberculosis (TB) in low-income and middle-income countries (LMICs) faces catastrophic household costs. We assessed the potential financial risk protection from introducing novel TB vaccines, and how health and economic benefits would be distributed across income quintiles.

**Methods:**

We modelled the impact of introducing TB vaccines meeting the World Health Organization preferred product characteristics in 105 LMICs. For each country, we assessed the distribution of health gains, patient costs and household financial vulnerability following introduction of an infant vaccine and separately for an adolescent/adult vaccine, compared with a ‘no-new-vaccine’ counterfactual. Patient-incurred direct and indirect costs of TB disease exceeding 20% of annual household income were defined as catastrophic.

**Results:**

Over 2028–2050, the health gains resulting from vaccine introduction were greatest in lower income quintiles, with the poorest 2 quintiles in each country accounting for 56% of total LMIC TB cases averted. Over this period, the infant vaccine was estimated to avert US$5.9 (95% uncertainty interval: US$5.3–6.5) billion in patient-incurred total costs, and the adolescent/adult vaccine was estimated to avert US$38.9 (US$36.6–41.5) billion. Additionally, 3.7 (3.3–4.1) million fewer households were projected to face catastrophic costs with the infant vaccine and 22.9 (21.4–24.5) million with the adolescent/adult vaccine, with 66% of gains accruing in the poorest 2 income quintiles.

**Conclusion:**

Under a range of assumptions, introducing novel TB vaccines would reduce income-based inequalities in the health and household economic outcomes of TB in LMICs.

**WHAT IS ALREADY KNOWN ON THIS TOPIC**

- Previous studies have shown that improved tuberculosis (TB) prevention and care can lower patient costs and reduce the number of TB-affected households experiencing catastrophic costs, and previous modelling has estimated the potential impact of TB vaccination on patient costs in country case studies.
- Survey evidence from high-burden countries has consistently demonstrated higher disease burden among poorer individuals, with TB prevalence in the lowest income quintile on average 2.3 times greater than estimated for the highest income quintile.
- TB patient cost surveys in high-burden countries have shown that TB patients experience high out-of-pocket and indirect costs and that these costs represent a greater share of annual household income in poorer income quintiles.

**WHAT THIS STUDY ADDS**

- This is the first study to examine the potential for new vaccines to reduce the number of households experiencing catastrophic costs due to TB, and how both these benefits and health gains are distributed across income quintiles.
- Across all modelled countries over 2028–2050, an adolescent/adult vaccine was projected to reduce TB incidence in the poorest quintile by 13.3 (95% uncertainty interval: 10.9–15.8) million (30% of total TB cases averted) and reduce the number of households experiencing catastrophic costs by 9.2 (7.5–11.0) million in the poorest quintile (40% of total cases of catastrophic costs averted) compared with the ‘no-new-vaccine’ baseline.

**HOW THIS STUDY MIGHT AFFECT RESEARCH, PRACTICE OR POLICY**

- Under a range of assumptions, new TB vaccines could be highly impactful and help narrow income-based disparities in the health and the economic consequences of TB for low-income and middle-income countries.

## Introduction

In 2021, 1.5 million individuals died of tuberculosis (TB).^1^ For individuals surviving TB, the disease episode represents an extended period of ill-health, which may lead to chronic disability.^2 3^ This burden of disease is not evenly distributed across populations between and within countries, with many TB risk factors—crowded living conditions, malnutrition, HIV and other factors that impair immune function—concentrated in poor and marginalised communities.^4^ Limited healthcare access in these communities also means that individuals developing TB may not receive prompt treatment, extending the duration and severity of disease. Nationally representative TB prevalence surveys conducted in high-burden countries have consistently demonstrated higher disease burden among poorer individuals, with TB prevalence in the lowest income quintile on average 2.3 times greater than estimated for the highest income quintile.^5–7^

In addition to the individual health effects, TB can have major economic consequences, especially for poor households.^8^ Individuals sick with TB may be less able to work, resulting in income losses. TB-associated healthcare typically involves substantial out-of-pocket costs for patients, despite government-provided TB treatment being free in many countries. Observational studies have shown that individuals with TB frequently make several care-seeking attempts before an accurate diagnosis is made,^9^ which involves additional costs. For poorer households, these costs can represent a substantial share of available income, increasing the risks of facing catastrophic costs.^8^ National survey evidence shows that one in two TB-affected households face costs exceeding 20% of household annual predisease income or expenditure.^10^

Bacillus Calmette-Guérin (BCG) vaccine is currently the only widely available TB vaccine. While routinely delivered to neonates in countries with a high burden of TB, BCG does not offer consistent protection against all forms of TB and in all age groups and is used to reduce the high case fatality rates associated with paediatric disease but with minimal impact on transmission or disease in older individuals.^11^ Consequently, several new TB vaccine candidates are in late-stage trials, and their successful development could create new opportunities to prevent TB, such as increased protection compared with BCG, the prevention of all forms of TB including drug-resistant TB and reactivation of TB and effectiveness in all age groups including HIV-infected persons.

Developing new safe, affordable and effective TB vaccines that can more rapidly reduce disease incidence and mortality is essential in the End TB Strategy approved by the World Health Assembly.^12^ However, the concentration of TB burden among poor people in low-income and middle-income countries (LMICs) with limited purchasing power for vaccines has created additional challenges in establishing the market for these vaccines and likely delayed vaccine development. Hence, new TB vaccines will likely only be developed if there is strong financial support from the global community in order to reach global development goals. Previous studies have shown that strengthened TB services can lower patient costs and reduce the number of TB-affected households experiencing catastrophic costs.^13 14^ While eliminating household catastrophic costs and promoting health equity are key elements of the End TB Strategy,^12^ there is little evidence on how new TB vaccines would contribute to these goals. In this study, we examined the potential for new vaccines to reduce the economic burden of TB on affected households and impact health inequalities. To undertake this study, we simulated the impact of vaccine products meeting the World Health Organization (WHO) preferred product characteristics (PPCs) for a new TB vaccine.^15^ Comparing these vaccination scenarios to a ‘no-new-vaccine’ baseline, we calculated the potential impact on patient-incurred direct and indirect costs, as well as the number of TB-affected households experiencing catastrophic costs in 105 LMICs over the period 2028–2050. We report how these outcomes—as well as the health benefits generated by vaccine introduction—would be distributed across income quintiles, to assess the potential for new TB vaccines to affect income-based inequalities in the health and economic burden of TB.

## Methods

### Vaccination scenarios

We evaluated an infant ‘preinfection’ vaccine (ie, efficacious for individuals uninfected at time of vaccination) with 80% efficacy to prevent disease targeting neonates and an adolescent/adult ‘preinfection and postinfection’ vaccine (ie, efficacious in all individuals without TB disease at time of vaccination) with 50% efficacy to prevent disease, as defined by the WHO PPCs for new TB vaccines. We assumed an average 10-year duration of protection and exponential waning. For the infant vaccine, this applied to infections acquired after vaccination. For the adolescent/adult vaccine, this applied to both new and prevalent infections. We assumed the infant vaccine would be delivered through the routine vaccination programme, and the adolescent/adult vaccine delivered through routine vaccination of 9 year olds and a one-time vaccination campaign for 10+ year olds. Based on consultation with global stakeholders, we assumed a coverage target of 85% for the infant vaccine (average coverage of diphtheria–tetanus–pertussis third dose for LMICs), 80% for routine delivery of the adolescent/adult vaccine and 70% for the adolescent/adult vaccination campaign,^16^ with equal coverage achieved within each income quintile in each country. We assumed countries would achieve linear scale-up to the specified coverage over 5 years, with starting coverage determined by average vaccination coverage of pneumococcal conjugate and rotavirus vaccines in the first year of introduction in LMICs,^17^ and introduce vaccination in country-specific years from 2028 to 2047, determined based on indicators for disease burden, immunisation capacity, classification of the country as an ‘early adopter/leader,’ lack of regulatory barriers and commercial prioritisation (online supplemental appendices S1–S2).^18^ We selected 2028 as the earliest country-specific introduction year to align with anticipated product availability following TB vaccine candidate trial completion, based on expert consultation and analysis (online supplemental appendix S1.7).

10.1136/bmjgh-2023-012466.supp1 Supplementary data

### Mathematical model

We developed a system of epidemiological and economic models, calibrated to demographic, epidemiological and health service data in 105 LMICs (accounting for 94.4% of TB incidence in LMICs^19^). Full epidemiological model details are described by Clark *et al* (summarised in online supplemental appendix S1).^18^ For each country, the model was stratified by income level (lower 40% vs upper 60% of population by household income), to reflect higher respiratory contact rates, greater TB risk factor prevalence and poorer healthcare access among lower-income groups (online supplemental appendix S1). This stratification approach aligns with existing equity frameworks that prioritise improvements for the poorest 40% of populations^20 21^ and facilitated further stratification of outcomes by income quintile (next section). The risk ratio of TB disease in the low-income stratum (vs high-income stratum) was calibrated to empirical data on income-based differentials in TB prevalence (online supplemental appendix S3).^18^

We simulated future TB-related outcomes in each modelled country for multiple scenarios over a 2028–2050 evaluation period. Each vaccine introduction scenario was compared with a ‘no-new-vaccine’ counterfactual—with current TB interventions (including provision of BCG vaccination to prevent severe disease in infants) assumed to continue in the future at their current coverage and quality (online supplemental appendix S1)—to estimate incremental changes in health service utilisation and TB-related health outcomes produced by vaccine introduction.

### Health outcomes

We assessed health outcomes as incident TB cases averted. To report results by household income quintile, we further stratified the modelled income strata to create five groups of equal population size (poorest/poorer/middle/richer/richest) with TB burden in these groups following the distribution of TB cases across income strata within published TB prevalence studies (online supplemental appendix S3). For each income quintile, we aggregated results across major country groupings (global, WHO region, World Bank income level^22^ and WHO high-TB burden grouping), to summarise the magnitude and *within-country* distribution of health gains.

### Costs incurred by TB-affected households

For each modelled country and income quintile, we calculated the costs incurred by TB patients and their households during the disease episode and applied these to the simulated number of TB cases by country, income quintile, scenario and year. Country-specific estimates of the patient costs per TB episode were derived from a meta-regression analysis of 20 nationally representative TB patient cost surveys.^10^ This study reported estimates for direct medical costs (medical products and services), direct non-medical costs (travel, accommodation, food, nutritional supplements) and indirect costs (income losses) incurred by TB patients, stratified by country and household income quintile, which we extracted for this analysis (online supplemental appendix S3). For each country and income quintile, we assumed that the per-patient costs of TB (in 2020 constant dollars) would not change in future years. For the base-case analysis, we assumed that individuals with TB disease who do not receive appropriate treatment (directly observed treatment, short-course) experience the same total per episode costs as those who receive appropriate treatment and examined alternative assumptions in sensitivity analyses. We assumed that a new TB vaccine would be provided free of charge and that households would incur no additional costs to receive the vaccine. We summarised results by country grouping and report costs in 2020 US dollars.

### Catastrophic costs

Following the WHO End TB target definition, we defined catastrophic costs as instances where the patient costs of TB disease—the sum of direct medical costs, direct non-medical costs and indirect costs—exceeded 20% of total annual income for the TB-affected household.^14 23–25^ For each country and income quintile, we assessed the number of TB-affected households experiencing catastrophic costs under each scenario, multiplying the probability of catastrophic costs per TB episode by the simulated number of TB cases by country, income quintile, scenario and year. Estimates of the probability of catastrophic costs per TB episode (stratified by country and income quintile) were derived from the meta-analysis of TB patient cost surveys^10^ used for patient cost estimates (online supplemental appendix S3). For each country and income quintile, we assumed that the probability of catastrophic costs for TB patients would not change in future years. We summarised catastrophic cost results by country income-level grouping.

### Distribution of benefits across countries and income strata

We undertook additional analyses describing how each major outcome (health gains, reductions in costs faced by patients, reductions in the proportion of households experiencing catastrophic costs) was distributed across the collective income gradient of the modelled countries. First, we ordered all country income quintiles (105 countries × 5 quintiles=525 unique groups) by average per capita gross domestic product (GDP) in 2020 purchasing power parity (PPP)-adjusted dollars. To do so, we obtained estimates of per capita PPP GDP and the fraction of total income held by each country income quintile, imputing missing values according to WHO region and income level group averages (eg, low-income countries in the African region).^26^ We multiplied these two terms and divided by the population fraction in each quintile (0.2) to obtain the average per capita PPP GDP for each quintile. We ranked all quintiles by average per capita PPP GDP and calculated the distribution of each study outcome across these quintiles. We summarised results graphically as well as via the Concentration Index, which quantifies the relative concentration of a given outcome in high-income or low-income groups. For a cumulative distribution representing incidence of an outcome in percentiles of a population ordered from poorest to richest (the ‘Concentration Curve’), the Concentration Index is defined as two times the area between the Concentration Curve and the line of equality (the 45° line, representing an equal distribution of the outcome across income groups). The index is defined in [−1, 1], with more positive (negative) values indicating greater concentration of the outcome in higher (lower)-income groups.^27^

### Sensitivity analysis

We propagated uncertainty in analytic inputs using a second-order Monte Carlo simulation (online supplemental appendix S3), which generated 1000 estimates for each outcome. We used this distribution of estimates to generate equal-tailed 95% uncertainty intervals for each study outcome, and to calculate partial rank correlation coefficients (PRCCs), which quantify the influence of individual parameters on study outcomes. We also examined the robustness of results to alternative analytic assumptions. First, compared with the base-case assumption of 50% efficacy for the adolescent/adult vaccine, we examined 75% efficacy conferred by this vaccine.^18^ Second, we examined an accelerated vaccine scale-up scenario whereby all countries introduce vaccination in 2025 and achieve instantaneous scale-up to the coverage target.^18^ Third, as there is substantial uncertainty around the costs incurred by patients who do not receive TB treatment, we re-estimated results under alternative scenarios that assumed costs for this group were 50% lower and higher, respectively, compared with individuals receiving TB treatment (vs the main analysis which assumed treated and untreated patients bore the same costs). Fourth, we examined alternative thresholds for defining catastrophic costs as 10% and 25% of total annual household income (vs 20% in the main analysis^1^). Fifth, we examined an alternative definition of catastrophic costs that only included direct medical costs (vs the main analysis which considered direct medical, direct non-medical and indirect costs). Finally, we reanalysed cost results applying a 3% discount rate (vs no discounting in the main analysis).

### Patient and public involvement

Patients and the public were not involved in this study.

## Results

### Overall impact of vaccine introduction

Compared with the ‘no-new-vaccine’ baseline, the base-case infant vaccine scenario averted costs borne by TB-affected households totalling US$5907 (95% uncertainty interval: US$5333–6533) million, including US$1036 (US$920–1143) million in direct medical costs, US$2264 (US$2027–2509) million in direct non-medical costs and US$2607 (US$2351–2896) million in indirect costs over the 2028–2050 study period in 105 LMICs (table 1). The adolescent/adult vaccine averted US$38 860 (US$36 594–42 461) million in total patient costs, including US$7252 (US$6758–7755) million in direct medical costs, US$14 987 (US$13 999–16 044) million in direct non-medical costs and US$16 620 (US$15 574–17 858) million in indirect costs (table 2). When we applied a 3% discount rate, total patient costs averted were US$3584 (US$3241–3953) million for the infant vaccine and US$26 352 (US$24 817–28 081) million for the adolescent/adult vaccine (online supplemental appendices S4–S5). We estimated 3.7 (3.3–4.1) million fewer households experiencing catastrophic costs with the infant vaccine and 22.9 (21.4–24.5) million fewer households with the adolescent/adult vaccine. Outcomes in the ‘no-new-vaccine’ baseline and associated percentage reductions achieved under the vaccination scenarios are presented in online supplemental appendices S6–S8.

**Table 1:** Costs borne by TB-affected households averted and number of households with catastrophic costs averted by infant tuberculosis vaccines (in millions)

Country grouping	Direct medical costs borne by TB-affected households averted	Direct non-medical costs borne by TB-affected households averted	Indirect costs borne by TB-affected households averted	Total costs borne by TB-affected households averted	Number of households with catastrophic costs averted
All countries	1036 (920–1143)	2264 (2027–2509)	2607 (2351–2896)	5907 (5333–6533)	3.69 (3.31–4.08)
High-TB burden*	814 (707–912)	1992 (1763–2236)	2112 (1876–2374)	4917 (4368–5497)	3.32 (2.94–3.70)
High-TB/HIV burden*	668 (566–764)	1728 (1504–1967)	1848 (1621–2097)	4245 (3702–4807)	2.84 (2.50–3.21)
High-MDR/RR-TB burden*	783 (677–882)	1783 (1561–2020)	1913 (1675–2172)	4479 (3939–5054)	2.89 (2.53–3.27)
	Income level†				
LIC	181 (145–219)	317 (270–369)	599 (487–725)	1096 (903–1299)	0.61 (0.52–0.73)
LMIC	699 (598–798)	1731 (1506–1976)	1554 (1360–1780)	3984 (3472–4543)	2.85 (2.50–3.25)
UMIC	156 (134–179)	217 (175–262)	454 (334–596)	827 (653–1030)	0.23 (0.17–0.29)
	World region				
AFR	483 (399–558)	1079 (920–1229)	1238 (1057–1425)	2800 (2405–3182)	1.78 (1.55–2.01)
AMR	27 (23.3–30.8)	25.8 (23–28.7)	27.9 (24.9–31.3)	80.8 (72.3–89.6)	0.01 (0.01–0.02)
EMR	190 (148–234)	302 (231–379)	513 (391–630)	1006 (779–1239)	0.51 (0.38–0.65)
EUR	21.9 (17.6–26.6)	10.5 (9–12.1)	14.9 (12.3–17.5)	47.2 (39.1–55.8)	0.008 (0.006–0.009)
SEAR	191 (144–250)	631 (489–800)	513 (403–655)	1335 (1030–1710)	1.01 (0.80–1.28)
WPR	122 (103–144)	216 (172–263)	300 (232–374)	639 (512–773)	0.37 (0.28–0.47)

All countries include 105 low-income and middle-income countries analysed.

Values in parentheses represent equal-tailed 95% credible intervals. Total costs included patient direct medical, direct non-medical and indirect costs (all undiscounted) over 2028–2050 in 2020 US dollars. Catastrophic costs are defined as instances where the total patient costs (direct medical, direct non-medical and indirect) incurred during an episode of TB disease exceeded 20% of total annual household income.

*High-TB, high-TB/HIV (HIV-associated TB) and high-MDR/RR-TB (multidrug/rifampicin-resistant TB) burden countries as defined by the WHO.

†LIC: gross national income (GNI) per capita of US$1085 or less; LMIC: GNI per capita of US$1086–4225; UMIC: GNI per capita of US$4256–13 205.^26^

AFR, African region; AMR, region of the Americas; EMR, Eastern Mediterranean region; EUR, European region; LIC, low-income country; LMIC, lower middle-income country; SEAR, Southeast Asian region; TB, tuberculosis; UMIC, upper middle-income country; WPR, Western Pacific region.

**Table 2:** Costs borne by TB-affected households averted and number of households with catastrophic costs averted by adolescent/adult tuberculosis vaccines (in millions)

Country grouping	Direct medical costs borne by TB-affected households averted	Direct non-medical costs borne by TB-affected households averted	Indirect costs borne by TB-affected households averted	Total costs borne by TB-affected households averted	Number of households with catastrophic costs averted
All countries	7252 (6758–7755)	14 987 (13 999–16 044)	16 620 (15 574–17 858)	38 860 (36 594–41 461)	22.9 (21.4–24.5)
High-TB burden*	5524 (5078–5956)	12 879 (11 895–13 896)	13 701 (12 699–14 820)	32 103 (29 894–34 544)	20.2 (18.7–21.8)
High-TB/HIV burden*	3807 (3449–4185)	10 719 (9803–11 698)	11 507 (10 547–12 597)	26 033 (23 851–28 389)	17.3 (15.8–18.8)
High-MDR/RR-TB burden*	5614 (5163–6103)	11 676 (10 733–12 687)	12 588 (11 605–13 676)	29 878 (27 590–32 293)	17.9 (16.4–19.4)
	Income level†				
LIC	876 (770–998)	1686 (1533–1849)	2842 (2494–3221)	5405 (4812–6034)	3.31 (3.02–3.62)
LMIC	4146 (3759–4547)	10 628 (9663–11 593)	9449 (8627–10 315)	24 223 (22 111–26 293)	17.3 (15.8–18.8)
UMIC	2230 (2002–2481)	2673 (2464–2899)	4329 (3774–4905)	9232 (8388–10 066)	2.25 (2.01–2.50)
	World region				
AFR	2126 (1933–2336)	5084 (4684–5500)	6597 (5968–7268)	13 808 (12 713–15 014)	8.52 (7.93–9.16)
AMR	415 (370–461)	530 (489–572)	513 (472–558)	1458 (1349–1568)	0.30 (0.28–0.32)
EMR	846 (717–988)	1410 (1170–1651)	2161 (1801–2557)	4418 (3734–5169)	2.35 (1.92–2.83)
EUR	428 (364–507)	219 (196–243)	300 (263–341)	947 (830–1078)	0.15 (0.13–0.17)
SEAR	1689 (1410–1997)	5638 (4829–6510)	4362 (3696–5071)	11 689 (9952–13 516)	8.94 (7.68–10.3)
WPR	1748 (1546–1967)	2105 (1905–2323)	2686 (2368–3034)	6539 (5907–7246)	2.63 (2.29–3.04)

All countries include 105 low- and middle-income countries analysed.

Values in parentheses represent equal-tailed 95% credible intervals. Total costs included patient direct medical, direct non-medical, and indirect costs (all undiscounted) over 2028–2050 in 2020 USD. Catastrophic costs are defined as instances where the total patient costs (direct medical, direct non-medical and indirect) incurred during an episode of TB disease exceeded 20% of total annual household income.

*High-TB, high-TB/HIV (HIV-associated TB), and high-MDR/RR-TB (multidrug/rifampicin-resistant TB) burden countries as defined by the WHO.

†LIC: gross national income (GNI) per capita of US$1085 or less; LMIC: GNI per capita of US$1086–4225; UMIC: GNI per capita of US$4256–13 205.^26^

AFR, African region; AMR, region of the Americas; EMR, Eastern Mediterranean region; EUR, European region; GDP, gross domestic product; LIC, low-income country; LMIC, lower middle-income country; SEAR, Southeast Asian region; TB, tuberculosis; UMIC, upper middle-income country; WPR, Western Pacific region.

### Health gains by quintile

As reported in our prior analyses,^18^ there were 6.7 (5.8–7.7) million TB cases averted by the infant vaccine and 44.0 (37.2–51.6) million cases by the adolescent/adult vaccine.

**Figure 1:**
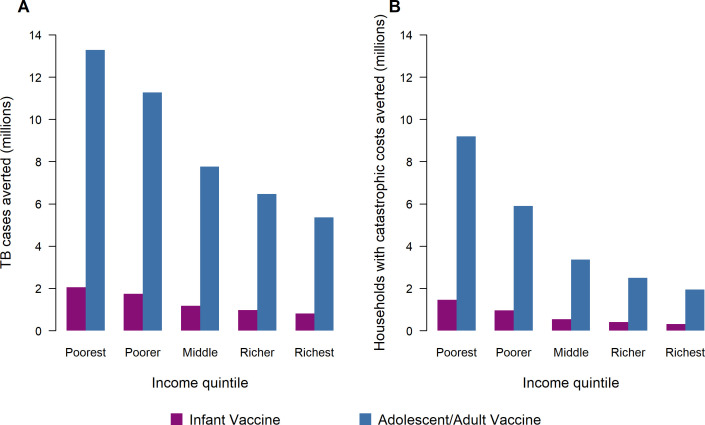
Tuberculosis (TB) cases averted (A) and number of households with catastrophic costs averted (B) by within-country income quintile comparing infant vaccine to adolescent/adult vaccine. Note: The total cost of a TB episode presented here included patient direct medical, direct non-medical and indirect costs over 2028–2050. Total costs borne by TB-affected households are categorised as ‘catastrophic’ if they exceed 20% of total household’s annual income.

**Figure 2:**
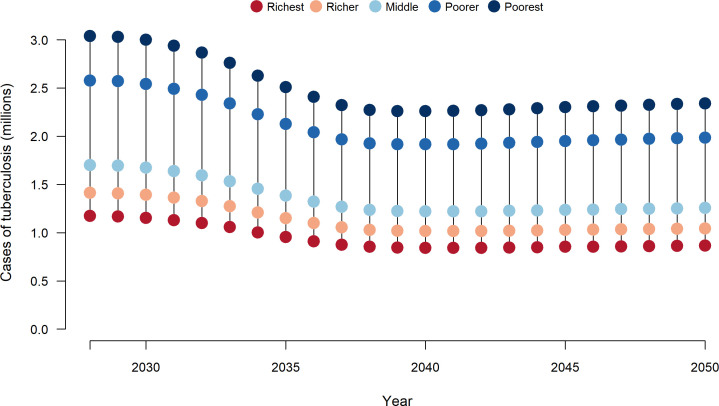
Cases of tuberculosis over time and by income quintile with delivery of adolescent/adult tuberculosis vaccines across 105 low-income and middle-income countries. Note: Country-specific vaccine introduction years from 2028 to 2047.

For both vaccine products, the number of TB cases averted by vaccine introduction was greatest in lower income quintiles. Across all modelled countries, the poorest 2 income quintiles accounted for 56% of total averted TB cases for both infant and adolescent/adult vaccination scenarios (figure 1A), with a Concentration Index calculated across five quintiles of −0.19. Figure 2 reports time trends in TB cases by income quintile for the adolescent/adult vaccination scenario, with relative differences in TB burden across income groups narrowing progressively over time (ie, a greater relative decline in the poorest compared with the richest income quintile), in addition to the absolute reductions experienced by all groups. The majority of health benefits (ie, TB cases averted by adolescent/adult vaccine introduction) accrue during 2028–2039 when the highest TB burden countries introduce vaccination and conduct a one-time mass vaccination campaign of all 10+ year olds.

### Averted patient costs by income quintile

The absolute reductions in TB patient costs resulting from vaccine introduction were weighted slightly towards higher income quintiles, with greater costs per episode of TB care incurred in these groups (online supplemental appendix S9) outweighing the greater reduction in TB cases in poorer quintiles. Across all modelled countries, the wealthiest 2 income quintiles accounted for 45% of total averted patient costs for the infant vaccination scenario (Concentration Index 0.06) and 46% for the adolescent/adult vaccination scenario (Concentration Index 0.07). When results were disaggregated by cost category, direct medical costs averted showed relatively equal distribution across quintiles (Concentration Index 0.03 and 0.04 for infant and adolescent/adult vaccines, respectively), while direct non-medical costs averted were concentrated in the poorest 2 quintiles (Concentration Index −0.09 and −0.08 for infant and adolescent/adult, respectively). The majority (56%) of indirect costs averted were concentrated in the wealthiest two quintiles (Concentration Index 0.20 and 0.21 for infant and adolescent/adult, respectively).

### Catastrophic costs averted by income quintile

The largest absolute reductions in the proportion of households facing catastrophic costs were in lower income quintiles within each country. Under each vaccination scenario, 66% of cases of catastrophic costs were averted in the poorest 2 quintiles (Concentration Index −0.31). Figure 1B shows the relative magnitude of cases of catastrophic costs averted across income quintiles. This gradient is steeper than for TB cases averted (figure 1A), indicating greater differences by quintile for catastrophic costs. The percentage of households experiencing catastrophic costs by country GDP per capita and by income quintile is presented in online supplemental appendix S10.

### Distribution of benefits across countries and income strata

**Figure 3:**
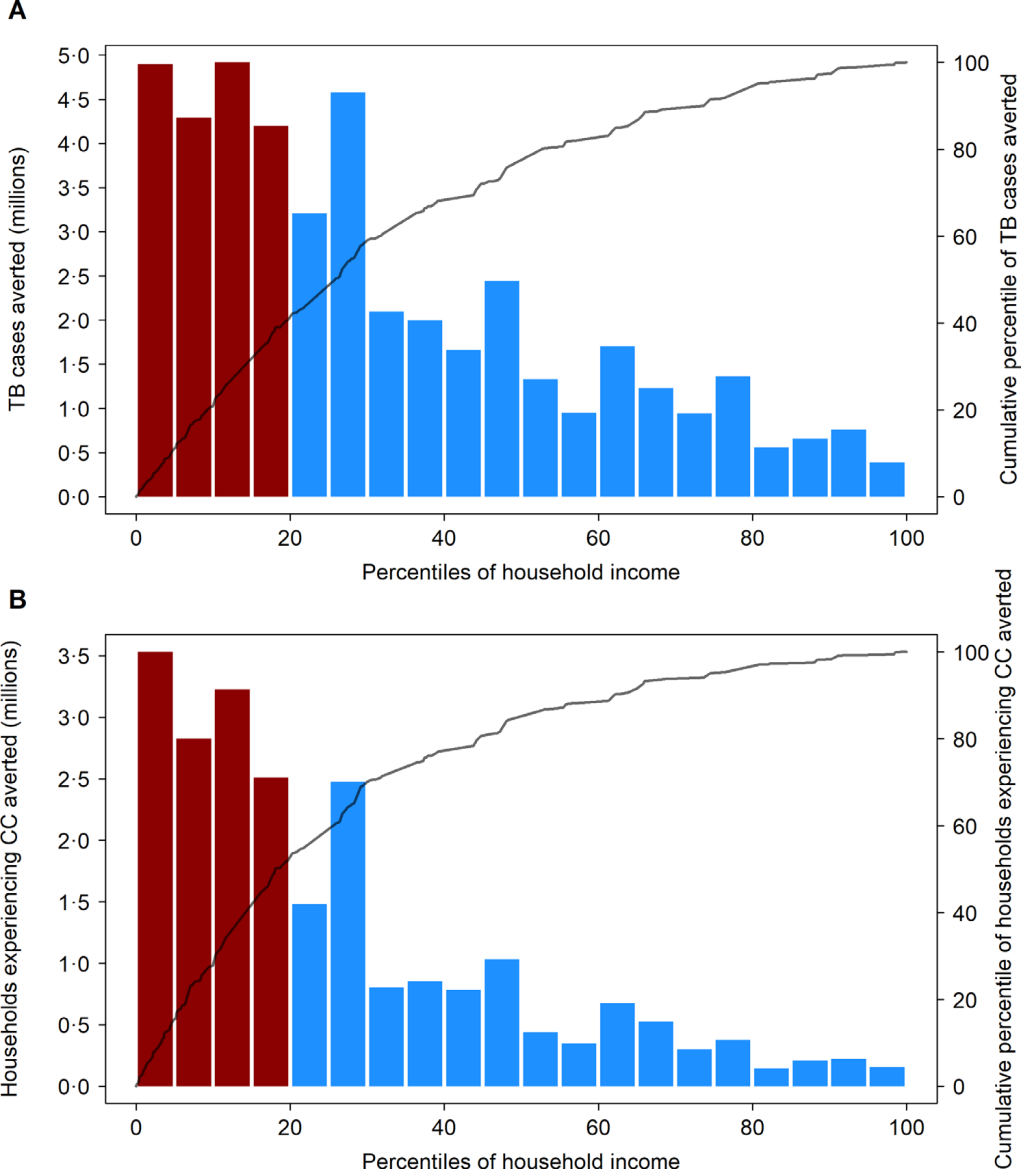
Distribution of tuberculosis cases averted (A) and number of households experiencing catastrophic costs averted over 2028–2050 (B) by an adolescent/adult vaccine across all modelled strata, ordered by household income. CC, catastrophic costs; GDP, gross domestic product; TB, tuberculosis. Bars defined by left-hand side y-axis; lines defined by right-hand side y-axis. Ordering of population by household income based on average 2020 per capita GDP in purchasing power parity (PPP) dollars, for each modelled stratum (525 total strata). Bars shaded red indicate the poorest 20% of modelled population by PPP GDP per capita.

Figure 3 shows the distribution of TB cases and catastrophic costs averted by household income across the combined population of the modelled countries over the 2028–2050 period for the adolescent/adult vaccine compared with the ‘no-new-vaccine’ counterfactual. For both outcomes, the benefits of vaccine introduction were concentrated in poorer households (poorest quintile shaded in red), with 18.3 (14.2–22.7) million TB cases projected to be averted in the poorest 20% of households (41% of total cases averted, Concentration Index −0.36) and 12.1 (9.4–15.0) million cases of catastrophic costs averted in the poorest 20% of households (53% of total cases of catastrophic costs averted, Concentration Index −0.48). Reductions in patient costs were also greater in the poorest households (online supplemental appendix S11), with US$11.8 (9.1–14.6) billion in cost savings projected to be averted in the poorest 20% of households (30% of total patient costs averted, Concentration Index −0.15).

### Sensitivity analyses

Compared with the assumed base-case efficacy of 50% for the adolescent/adult vaccine, an assumption of 75% efficacy had greater impact, averting 33.4 (31.2–35.8) million cases of catastrophic costs (online supplemental appendix S12). Compared with the base-case scenario, the accelerated scale-up scenario had greater household economic impact for both infant and adolescent/adult vaccination (online supplemental appendices S13–S14), with 8.8 (8.0–9.8) million cases of catastrophic costs averted for the infant vaccine and 33.9 (31.7–36.3) million for the adolescent/adult vaccine. Assuming that individuals with TB who do not receive appropriate treatment experience 50% lower costs compared with treated individuals reduced the number of households with averted catastrophic costs, to 2.8 (2.5–3.1) million for the infant vaccine and 18.7 (17.6–20.0) million for the adolescent/adult vaccine (online supplemental appendices S15–S18). Assuming these individuals experience 50% higher costs compared with treated individuals increased the number of households with averted catastrophic costs, to 4.2 (3.8–4.7) million for the infant vaccine and 25.4 (23.6–27.2) million for the adolescent/adult vaccine (online supplemental appendices S19–S22). Using alternative definitions of catastrophic costs, such as instances where patient costs exceed 10% of annual household income (vs 20% in the main analysis), resulted in approximately 40% more cases of catastrophic costs averted in infant and adolescent/adult vaccine scenarios (online supplemental appendix S23). A higher threshold for defining catastrophic costs (ie, >25% of annual household income), resulted in approximately 14% fewer cases of catastrophic costs averted in infant and adolescent/adult vaccine scenarios (online supplemental appendix S24). When we restricted the definition of catastrophic costs to only consider direct medical costs (vs the sum of direct medical, direct non-medical and indirect costs in the main analysis), we estimated approximately 84% fewer cases of catastrophic costs averted compared with the main analysis, for both vaccine products (online supplemental appendices S23–S24). When we applied a 3% discount rate to total (direct and indirect) costs incurred in future years (vs no discount rate in the main analysis), total patient costs averted were US$3584 (US$3241–3953) million and US$26 352 (US$24 817–28 081) million for infant and adolescent/adult vaccines respectively, 39% and 32% less than in the main analysis (online supplemental appendices S4–S5).

Online supplemental appendix S25 shows PRCCs reflecting the relative influence of model parameters on the number of cases of catastrophic costs projected to be averted by the adolescent/adult vaccine. This analysis shows the rate of progression from subclinical to clinical TB disease (PRCC=−0.559), the rate of treatment initiation (PRCC=−0.472) and the rate of fast progression to TB disease (PRCC=0.425) were the most influential parameters for this outcome. Online supplemental appendix S26 shows PRCCs for the number of cases of catastrophic costs averted by the infant vaccine, which were similar to the PRCC results for the adolescent/adult vaccine.

## Discussion

In this study, we estimated the potential impact of new TB vaccines on income-based disparities in the health and economic consequences of TB in LMICs. Both infant and adolescent/adult vaccines were projected to reduce disparities in TB disease burden, with the adolescent/adult vaccine estimated to have greater absolute impact. Across all modelled countries over 2028–2050, an adolescent/adult vaccine was projected to reduce the number of incident TB cases in the poorest quintile by 13.3 (10.9–15.8) million (30% of total TB cases averted) and reduce the number of households experiencing total costs of care exceeding 20% of household income (ie, catastrophic costs) by 9.2 (7.5–11.0) million in the poorest quintile (40% of total cases of catastrophic costs averted) compared with the ‘no-new-vaccine’ baseline.

The concentration of vaccine impact in lower-income groups results from two features of TB in LMICs. First, current TB burden is concentrated in low-income groups. Individuals in poor households are more likely to be infected, have a greater concentration of risk factors for developing TB disease and are more likely to die from TB if it occurs.^1^ These differences are rooted in the socioeconomic determinants of TB and have motivated a global TB strategy that emphasises vulnerable groups and strengthening social protection.^28 29^ Developing an effective TB vaccine is a key component of this strategy. Second, poor households are uniquely vulnerable to health shocks, which can lead to economic hardship and medical debt. In the meta-analysis of TB patient cost surveys^10^ used for this analysis, the fraction of patients experiencing catastrophic costs was higher for each successive income quintile from richest to poorest.

In addition to reporting the distribution of heath and economic benefits of TB vaccination within each country, we also analysed the distribution of benefits across the combined income gradient of the 105 modelled countries. The results from this analysis are qualitatively similar to the within-country analyses,^10^ though with greater concentration of benefits within poorer groups for each outcome (more negative Concentration Index values), highlighting the concentration of potential benefits within poor countries, as well as within poor groups within each country. For all outcomes, the benefits of vaccination were spread over the evaluation period, such that discounted cost outcomes (an outcome that downweights costs averted many years in the future) were estimated to be 30%–40% smaller than undiscounted cost outcomes. This difference reflects the staggered vaccine introduction timeline as well as the delay between vaccine introduction and subsequent reductions in TB incidence.

This analysis has several limitations. First, the characteristics of a new TB vaccine, once available, may differ from the scenarios we examined, which were based on the WHO PPCs.^15^ These PPCs represent preferred vaccine attributes to deliver public health impact, but a final product may differ in terms of effectiveness or duration of protection.^18^ Changes in vaccine performance would have implications for the results of this analysis, as demonstrated in the sensitivity analyses. Second, we made assumptions about vaccine introduction based on expert opinion and historical vaccine introduction patterns. We did not consider all possible introduction scenarios, such as vaccine coverage targets and scale-up trends by country or income quintile, but demonstrated the potential value of novel TB vaccines according to specified characteristics. As demonstrated in the sensitivity analyses, the impact of vaccination will depend on how aggressively countries scale-up a new vaccine, with accelerated introduction increasing the magnitude of impact.^18^ Third, we assumed random mixing between the low access-to-care and high access-to-care groups in this analysis. If mixing is assortative and/or more concentrated in particular quintiles, interventions reducing TB incidence in one group would produce smaller changes in new infections in the other group, compared with an assumption of random and uniform mixing. We did not examine such situations in this analysis. Fourth, we assumed that vaccine coverage would be the same across all income quintiles in each country. However, the evidence for routine immunisation delivery shows heterogeneity in coverage between income groups, with a trend towards lower coverage in poorer income quintiles.^30^ Efforts to lower access barriers in low-income groups may be needed to prevent inequalities in TB vaccine coverage. Related to this, we assumed vaccines would be provided free of charge. Requiring payment for vaccination would likely reduce uptake, particularly within low-income groups. Fifth, there is substantial uncertainty around the cost faced by individuals not receiving treatment. In the base-case analysis, we assumed that untreated individuals experienced the same costs as treated individuals, based on limited evidence highlighting costs faced by these individuals^31^ and examined alternative assumptions in sensitivity analysis. We also examined alternative thresholds for defining catastrophic costs as a percentage of total annual household income in sensitivity analysis. Sixth, we assumed that the patient costs of each TB episode (in constant US dollars) would be unchanged over the 2028–2050 study period; and that real incomes would also be fixed. If the patient costs per TB episode were to decline, or real incomes rise, this would reduce the number of households experiencing catastrophic costs due to TB. Finally, we assumed that TB trends in the ‘no-new-vaccine’ baseline would follow their historical trajectory (online supplemental appendix S1), consistent with ongoing provision of TB services at current quality and coverage levels. If there were aggressive scale-up of currently available interventions (as envisaged by the recent global TB strategy), this would reduce the incremental impact of a new vaccine.^18^ Similarly, if the novel vaccinations replaced current services (BCG vaccination, TB preventive therapy), this could also lead to smaller incremental impact. Future analyses are needed to compare vaccination strategies to other TB service improvements and take into account the health system capacity of each country to inform more realistic delivery scenarios.

Policy-makers consider several issues when prioritising health interventions; one of the most important for diseases of poverty such as TB is impact on health equity.^32^ This manuscript demonstrates that under a range of assumptions, new TB vaccines could be highly impactful and help narrow income-based disparities in the health and the economic consequences of TB for LMICs, helping achieve the WHO End TB Strategy goals, make substantial progress towards achieving Universal Health Coverage and sustainable development goal targets (eg, eradicating poverty inSustainable Development Goal (SDG) 1, hunger in SDG 2, promoting decent work and growth in SDG 8 and good health and well-being in SDG 3). In addition to evidence on epidemiological impact^18^ and cost-effectiveness,^33^ these results confirm the broad range of benefits that could be achieved by an effective TB vaccine. To achieve these benefits, countries will need to commit to rapid introduction once an effective vaccine is approved, achieve high population coverage and prevent differentials in vaccine access for poor and marginalised groups. Doing so will require sustained political and financial commitments by affected countries and international partners, as well as implementation research on approaches to eliminate access barriers during vaccine introduction. While major challenges remain, successful development and introduction of a new TB vaccine has the potential to accelerate the elimination of a disease that has represented one of the greatest health threats for poor households for millennia.

## Data Availability

Data are available in a public, open access repository. The authors confirm that all data underlying the findings are fully available without restriction. Analytic code is available at https://doi.org/10.5281/zenodo.6421372.
