# Supplementary material for: The potential impact of novel tuberculosis vaccines on health equity and financial protection in low-income and middle-income countries
Source: BMJ Glob Health. 2023 Jul 12;8(7):e012466. doi: 10.1136/bmjgh-2023-012466 (PMC10347450; doi:10.1136/bmjgh-2023-012466)
Supplement: Supplementary data [file bmjgh-2023-012466supp001.pdf]

Appendix S1. Epidemiological methods

The subsequent pages provide relevant details and methods regarding the underlying epidemiological model and vaccine delivery scenarios from Clark et al., 2023.[1]

S1.1. Tuberculosis natural history structure

The core natural history model is specified in Figure A. Those with no previous exposure or infection with *Mtb* [Uninfected-Naive (U_N)] could become infected at rate λ_j and progress to an Infection-Fast (I_F) class following initial infection. From Infection-Fast, three possible pathways were possible: (i) Fast progression to Subclinical Disease (D_S), where individuals are infectious with a reduced infectiousness compared to clinical tuberculosis, but display no symptoms of tuberculosis disease;[2] (ii) self-clearance to Uninfected-Cleared (U_C), where individuals are no longer infected with *Mtb* and therefore are not at risk of progression to tuberculosis disease without reinfection;[3] or (iii) continue to remain latently infected with a risk of reactivation and progression to disease, albeit at a lower rate than Infection-Fast, by transitioning to the Infection-Slow (I_S) class. Those in the Infection-Slow class could self-clear to the Uninfected-Cleared class, be reinfected and return to the Infection-Fast class or reactivate their infection and progress to Subclinical Disease.

Once in the Subclinical Disease class, individuals could naturally cure (without treatment) to the Resolved (R) class, or progress to Clinical Disease (D_C), where individuals are infectious and display symptoms of tuberculosis disease. Treatment initiation from Clinical Disease to On-Treatment (T) began in 1960 and increased following a sigmoid curve to 2019, with average treatment duration assumed to be six months.[4, 5] Treatment completions transitioned to the Resolved class and treatment non-completions returned to Clinical Disease. Deaths occurring on-treatment and in clinical disease counted toward the total number of tuberculosis deaths during the year. Those with clinical disease could also naturally cure to the resolved class. Individuals in the Resolved class could be reinfected or relapse to Subclinical. We assumed that the infection and resolved classes are partially protected against reinfection.[6, 7] In those who have self-cleared, we assumed the level of protection against reinfection is half of the protection against reinfection for the infection and resolved classes. Age was modelled in single years from ages 0 to 79 and aggregated into two categories for ages 80 to 89, and ages 90 to 99. Births and ageing occurred at the beginning of each year.

S1.2. HIV and ART structure description

To account for the influences of human immunodeficiency virus (HIV) and antiretroviral therapy (ART) on the risk of infection with *Mtb* and progression to tuberculosis disease,[6, 8] we implemented an HIV structure (shown in Figure B) composed of 3 compartments: HIV uninfected [HIV0], people living with HIV (PLHIV) not on ART [HIV1], and PLHIV on ART [ART]. HIV uninfected individuals were diagnosed with HIV and moved from the HIV0 compartment to the HIV1 compartment with rate λ_H. Within the HIV1 compartment, there is a higher risk of tuberculosis progression and an increased tuberculosis mortality rate compared to the HIV0 compartment. PLHIV are initiated on treatment with ART from HIV1 following a sigmoid trend.

The increases in tuberculosis mortality rate and tuberculosis progression are reduced while in ART compared to HIV1, but still higher than in HIV0. ART also reduces the HIV mortality rate.

The separate stratum was included to dynamically model the tuberculosis-HIV co-epidemic if the proportion of tuberculosis cases among people living with HIV (PLHIV) was greater than or equal to 15%, and if the HIV prevalence in the country was greater than 1%. Countries incorporating the additional HIV structure are listed in Table C.

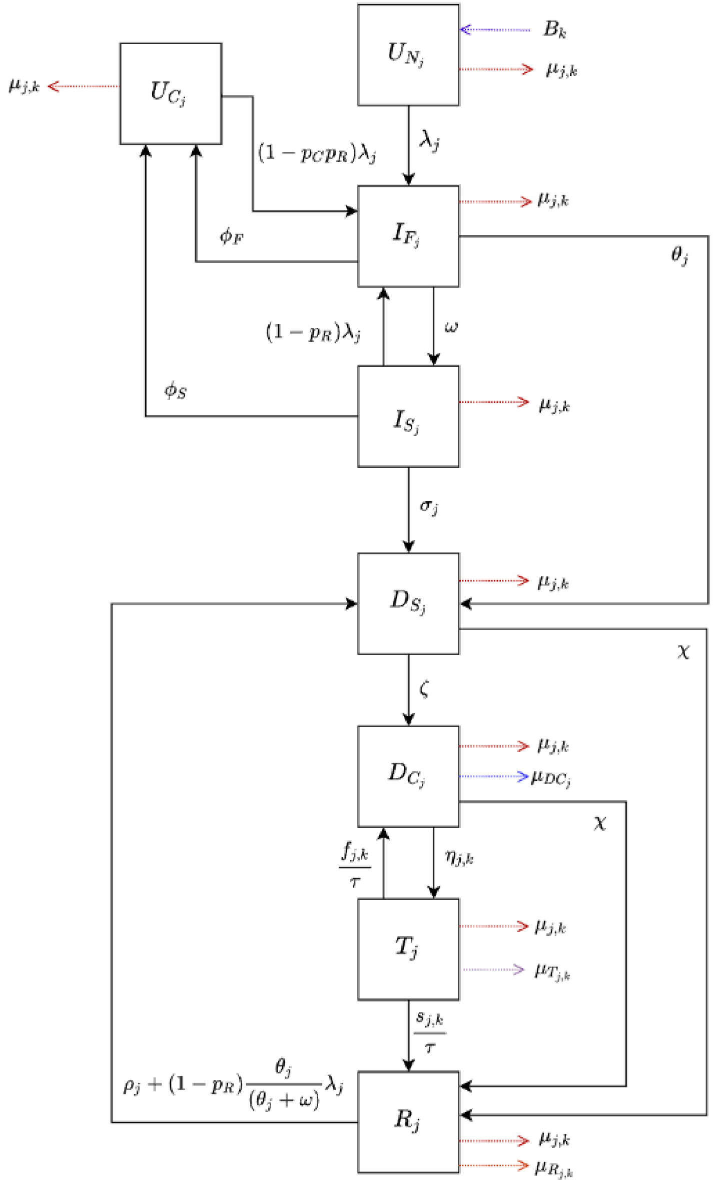

Figure A. Tuberculosis natural history model

Abbreviations: D_C = Clinical Disease; D_S = Subclinical Disease; I_F = Infection-Fast; I_S = Infection-Slow; R = Resolved; T = On-Treatment; U_C = Uninfected-Cleared; U_N = Uninfected-Naïve.

Portnoy A, *et al. BMJ Glob Health* 2023; 8:e012466. doi: 10.1136/bmjgh-2023-012466

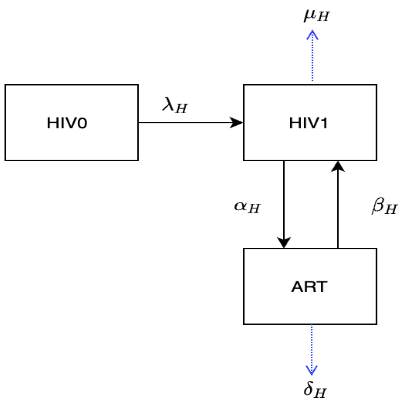

Figure B. HIV and ART structure.
Abbreviations: ART = People living with HIV on ART; HIV0 = HIV uninfected; HIV1= People living with HIV not on ART.

Table C. Countries incorporating the HIV structure with their corresponding HIV prevalence and proportion of tuberculosis cases among PLHIV.

Country	HIV Prevalence (%)	Proportion of tuberculosis cases among PLHIV (%)
Botswana	16.5	48.6
Central African Republic	2.1	25.4
Côte d'Ivoire	1.7	17.5
Cameroon	2.0	26.8
Gabon	2.3	32.8
Ghana	1.1	20.8
The Gambia	1.2	17.7
Guinea-Bissau	2.1	31.3
Equatorial Guinea	4.8	26.5
Guyana	1.1	19.0
Kenya	2.9	26.2
Lesotho	16.0	61.6
Mozambique	7.2	33.8
Malawi	5.9	46.6
Namibia	8.4	32.5
Rwanda	1.8	21.1
Eswatini	17.4	60.1
Togo	1.5	16.2
Tanzania	2.9	23.6
Uganda	3.4	39.0
South Africa	12.8	58.0
Zambia	6.7	46.2
Zimbabwe	9.6	59.8

S1.3. Access to care structure

The access to care dimension is incorporated to allow for the negative correlation between tuberculosis burden and health care access to prevent the overestimation of vaccine impact, as well as to facilitate analyses of equity implications of vaccine introduction. The access to care dimension contains 2 classes: high-access-to-care, representing the top income 3 quintiles (60% of the population) and low-access-to-care, representing the bottom 2 income quintiles (40% of the population). We assumed that there was no transition between the high- and low-access-to-care classes, as well as assuming random mixing between the high-access-to-care and low-access-to-care classes.

To constrain relative burden between access-to-care classes, we calibrated the tuberculosis prevalence ratio in the high-access-to-care class to the low-access-to-care class in 2019. The calibration target, 0.674, was calculated as a weighted average from eleven studies, with lower and upper bounds (0.575–0.801) representing the 25th and 75th percentiles of the datasets (Table D).[9-19]

Table D. TB prevalence study data.

Source	Country	Prevalence rate ratio of upper 60% vs. lower 40% of population by socioeconomic status	Weight
[11]	Bangladesh	0.394	1
[14]	India	0.386	1
[15]	India	0.467	1
[17]	Kenya	0.588	1
[16]	Malawi	0.867	1
[16]	Mongolia	0.716	1
[16]	Myanmar	0.807	1
[16]	Philippines	0.755	0.5
[17]	Philippines	0.608	0.5
[13]	Rwanda	1.081	1
[16]	Rwanda	0.774	1
[9]	South Africa	0.486	1
[18]	South Africa	0.896	1
[16]	Tanzania	0.648	1
[10]	Vietnam	0.701	1
[19]	Vietnam	0.799	1
[16]	Vietnam	0.672	1
[12]	Zambia	0.534	0.7
[16]	Zambia	1.312	0.7
[18]	Zambia	0.728	0.7

To incorporate access to care into our model, we assume that the differences in tuberculosis burden between classes are due to differences in the force of infection, the rate of care-seeking (i.e., tuberculosis treatment initiation), and the rate of tuberculosis progression. We assume relative to the low-access-to-care stratum, the high-access-to-care stratum has a reduced force of infection per contact, an increased rate of treatment initiation, and a reduced rate of tuberculosis progression. Differential burden was implemented by introducing a new parameter p_E, such that $p_E \in [0, 1]$ for the high-access-to-care stratum and $p_E = 0$ for the low-access-to-care stratum. This parameter was included within the model natural history structure as described in Table E. This new parameter was fitted during calibration.

Table E. Implementing the access-to-care parameter

	**Access-to-Care**

**Force of infection**	$p_T \times \sum_{y=1}^{n_{ygroups}} (1-p_E) \times C[m,y] \times \left(\frac{(1-ep)(TD_{C_y}+rTD_{S_y})}{N_y}\right)$
**Treatment Initiation Rate**	$\frac{\eta_{j,k}}{(1-p_E)}$
**Rate of Tuberculosis Progression**	$(1-p_E) \times \theta_j$<br>$(1-p_E) \times \sigma_j$<br>$(1-p_E) \times \rho_j$

S1.4. Calibration methodology

The model was fitted to epidemiologic calibration targets using history matching with emulation, implemented using the hmer R package.[20, 21] If countries were unable to find at least 1000 fully fitted parameter sets using this method, they were subsequently assessed using an Approximate Bayesian Computation using Markov Chain Monte Carlo method (ABC-MCMC). ABC-MCMC was conducted using the easyABC package in R, modified by Sebastian Funk, Gwenan Knight, and the Tuberculosis Modelling group at LSHTM for adaptive sampling and to accept seeded parameter values.[20, 22] We used parameter sets with the maximum number of targets fitted using history matching with emulation as a starting seed, with the ABC-MCMC algorithm continuously adapting using the last 1000 points and the noise factor set to 0.0001.

Analysis was performed on 105 countries from the 135 total low- and middle-income countries identified based on 2019 World Bank Income groups. There were 20 countries excluded from the initial calibration attempt due to missing crucial data required to attempt calibration, and 10 countries which were unable to be calibrated (could not find a parameter set that matched all targets using both history matching with emulation as well as ABC-MCMC). Reasons for exclusion from the final list of calibrated countries are provided in Table F.

Table F. Reasons for exclusion from the final list of calibrated countries.

Country	Reason for Exclusion
Algeria	Did not calibrate
American Samoa	Missing multiple critical epidemiological data for calibration, no contact matrices available
Belize	No case notification or incidence data for children
Bosnia and Herzegovina	Did not calibrate
Cabo Verde	Did not calibrate
Comoros	No case notification data
Democratic Republic of the Congo	No case notification data by age
Republic of the Congo	No population estimates
Democratic People's Republic of Korea	Missing multiple critical epidemiological data for calibration
Djibouti	No case notification data by age
Dominica	Missing multiple critical epidemiological data for calibration, no contact matrices available
Guinea-Bissau	Did not calibrate
Guyana	Did not calibrate
Federated States of Micronesia	Missing multiple critical epidemiological data for calibration, no contact matrices available
Grenada	Missing multiple critical epidemiological data for calibration, no contact matrices available
Haiti	Missing 2020 contact matrix
Jamaica	Did not calibrate
Kiribati	Missing 2020 contact matrix
Kosovo	Missing multiple critical epidemiological data for calibration
Lebanon	Missing 2020 contact matrix
Marshall Islands	Missing multiple critical epidemiological data for calibration, no contact matrices available
North Macedonia	Did not calibrate

Portnoy A, *et al. BMJ Glob Health* 2023; 8:e012466. doi: 10.1136/bmjgh-2023-012466

Samoa	No case notification or incidence data for children
Somalia	No contact matrices available
St. Lucia	No case notification or incidence data for children
St. Vincent and the Grenadines	Did not calibrate
Tonga	Did not calibrate
Turkmenistan	Did not calibrate
Tuvalu	No contact matrices available
West Bank and Gaza	Missing multiple critical epidemiological data for calibration

S1.5. Vaccine profile

The vaccine profile for an adult/adolescent vaccine and infant vaccine were based on the WHO Preferred Product Characteristics for New Tuberculosis vaccines,[23] and are outlined in Table G below.

Table G. WHO Preferred Product Characteristics for New Tuberculosis Vaccines.

Vaccine	Host infection status at time of vaccination required for efficacy	Effect type	Vaccine efficacy	Duration of protection
Adolescent / Adult	Pre- and post-infection	Prevention of disease	50%	Lifelong
				10 years
Infant	Pre-infection	Prevention of disease	80%	Lifelong
				10 years

Vaccine efficacy was assumed to be the same in both PLHIV and HIV-naïve recipients in countries incorporating the HIV structure, and in both younger age groups and older adults. The vaccine was assumed to have the same impact on preventing drug-susceptible and drug-resistant tuberculosis as specified in the WHO PPCs.[23] As we were modelling a prevention of disease vaccine, there was no direct impact on *Mtb* transmission or the force of infection.

We assumed duration of protection was 10 years on average, in addition to a sensitivity analysis with lifelong duration of protection. The shape of waning immunity was modelled as an exponential distribution, based on similar shapes for waning vaccine immunity of BCG[24] and other vaccines.[25, 26]

S1.6. Vaccine delivery scenarios

The infant vaccine was implemented in two scenarios, and, separately, the adolescent/adult vaccine was implemented in three scenarios. The *Basecase* and *Accelerated Scale-up* scenarios included routine single-dose neonatal vaccination for the infant vaccine (85% coverage), and routine single-dose vaccination of 9-year-olds (80% coverage) with a one-time vaccination campaign for ages ten and older (70% coverage) for the adolescent/adult vaccine. The *Routine Only* scenario (adolescent/adult vaccine only) was introduced through routine 9-year-old vaccination only (i.e., no campaign). Specifics of the infant and adolescent/adult vaccine scenarios are provided in Table H.

Table H. Vaccine scenarios for the infant and adolescent/adult vaccines.

Characteristics	Infant Vaccine Scenarios		Adolescent/Adult Vaccine Scenarios		
	Basecase	***Accelerated Scale-up***	***Basecase***	***Accelerated Scale-up***	***Routine Only***
**Ages Targeted**	*Neonatal:* Routine	*Neonatal:* Routine	*Age 9:* Routine<br><br>*Ages 10+:* One-time vaccination campaign over 5 years	*Age 9:* Routine<br><br>*Ages 10+:* One-time vaccination campaign in 2025	*Age 9:* Routine
**Introduction Year**	Country-specific	2025	Country-specific	2025	Country-specific
**Vaccine Rollout Trend**	5-year linear scale-up to coverage	Instant scale-up to coverage	5-year linear scale-up to coverage	Instant scale-up to coverage	5-year linear scale-up to coverage
**Target Coverage *(Low/Med/High)***	75% / 85% / 95%		Age 9: 70% / 80% / 90%<br>Ages 10+: 50% / 70% / 90%		

S1.7. Country-specific introduction years

In the *Basecase* and *Routine Only* scenarios, vaccines were introduced in country-specific introduction years between 2028 and 2047. The year 2028 was selected as the earliest country-specific introduction year to align with the anticipated completion and availability of results from TB vaccine candidate trials based on expert consultation and analysis. Country-specific introduction years were calculated for all 135 LMICs based on the 2019 World Bank Income groups. To calculate the specific year of introduction, countries were divided into two general categories: those procuring with support from Gavi, the Vaccine Alliance, and those self-procuring. Determination of country status was based on eligibility information posted on Gavi's website.[27] Countries transitioning from Gavi support are able to benefit from Gavi pricing and incremental financing for a period of 5–10 years. For countries that have already initiated the period of transition by 2019, this window will have largely ended by the time of tuberculosis vaccine availability through Gavi. As such, these countries were categorised as self-procuring countries. Countries that have not yet commenced transition, including India and Nigeria, were categorised as Gavi supported countries, given the long grace period post-commencement of transition. For more information, please see Gavi, https://www.gavi.org/types-support/sustainability/transition (retrieved December 1, 2020).

Through a consultative process with experts from WHO, Gavi, PATH, PDVAC, CHAI, and industry partners, factors influencing likelihood of being an early or late adopter were identified for both Gavi and self-procuring countries. Identified factors include disease burden, immunization

capacity, and early adopter status. Country-specific registration timelines and commercial prioritization were also deemed important determinants of introduction timing for self-procuring countries.

Additional factors for Gavi countries: For countries procuring through Gavi, timelines for introduction are also influenced by Gavi processes. Prior to offering a new vaccine, Gavi requires that products be licensed, included in Gavi's Vaccine Investment Strategy, reviewed by SAGE, recommended in a WHO position paper, WHO prequalified, and approved for procurement by Gavi (Table I). In addition, time for country application processing, contracting, and delivery must be factored. Through consultations, it was determined that a baseline time of roughly two years post licensure would be needed for Gavi processes prior to first country introduction, assuming several steps advance in parallel.

Table I. Timelines for Gavi processes post licensure.

	**Cumulative additional time (years)**		
**Activities post licensure**	**Low End**	**High End**	**Average**
WHO PQ	0.25	1.00	0.63
SAGE Policy Review & WHO Position Paper	0.25	0.50	0.38
Gavi Decision	0.25	0.50	0.38
National review & Country applications	0.25	0.75	0.50
Contracting & delivery	0.25	0.50	0.38
**Years**	**1.25**	**3.25**	**2.25**

Weight of criteria, indicators, and scoring: Differential weight was assigned to criteria based on their relative impact on the order of country adoption. This weight varied for self-procuring and Gavi countries (Table J).

Table J. Weight of criteria influencing order of country adoption.

Criteria	Self-procuring countries	Gavi countries
Disease burden	30%	45%
Immunization capacity	15%	30%
Early adopter/leader	15%	25%
Lack of regulatory barriers	15%	NA
Commercial prioritization	25%	NA

The following indicators were used to measure each of the variables identified in Table K.

Table K. Indicators of criteria influencing order of country adoption.

Criteria	Indicator
**Disease burden**	Tuberculosis incidence
**Immunization capacity**	Proportion receiving 3 doses of DPT3 among infants 1 years of age (The percent of infants receiving 3 doses DPT3 is commonly used as a proxy for assessing immunization infrastructure)
**Lack of regulatory barriers**	Signatories to WHO PQ or SRA collaborative registration scheme<br>Lack of requirements for additional local clinical trial data
**Early adopter/leader**	Time to policy adoption of universal Xpert MTB/RIF screening for presumed tuberculosis cases<br>Time to adoption of HPV
**Commercial prioritization**	
Ability to finance vaccines	GDP per capita
Political will to address tuberculosis	Spending per tuberculosis case
Market potential	Population

To standardize across these varied metrics, a point value ranging from 1–5 per criteria was assigned, with a score of 1 correlating with an earlier adopter and score of 5 correlating with a later adopter.

*Continuous variable*s such as disease burden or population were divided into quintiles. Those in the highest quintile were assigned a score of 1, those in the second highest quintile received a score of 2, and so forth. *Categorical variables* such as registration or early adopter status were scored based on whether countries met fixed criteria. For instance, countries that are signatories of WHO PQ or SRA collaborative registration schemes were assigned a score of 1. Those that are not signatories and have requirements for additional clinical trial data in local populations received a score of 5.

Scores were then weighted as reflected in Table J and aggregated into a composite score to determine countries' relative position in the queue of introductions.

Assumptions for the pace of introduction—i.e., how many countries per year would introduce the product and what the scale up curve might look like— was informed with data from pneumococcal vaccine (PCV) scale-up.[28] The percent of countries adopting each year (year 1 to year 12) for PCV was calculated. These annual percentages were then applied to tuberculosis vaccine scale up (based on a total n=135 countries: 78 self-procuring countries and 57 Gavi countries). The first year of tuberculosis vaccine scale up was estimated to be 2028, with Gavi countries following a similar scale up trajectory but delayed by two years due to required Gavi lead time for processing new vaccines (Table I). Because PCV data is only available for 12 years, data was extrapolated for years 13 to 20 of tuberculosis vaccine roll out at a steady state. Country introduction timelines were adjusted—where applicable—to group countries with the same composite score in the same year of adoption. The cumulative number of countries introducing the vaccine by year is shown in Figure L.

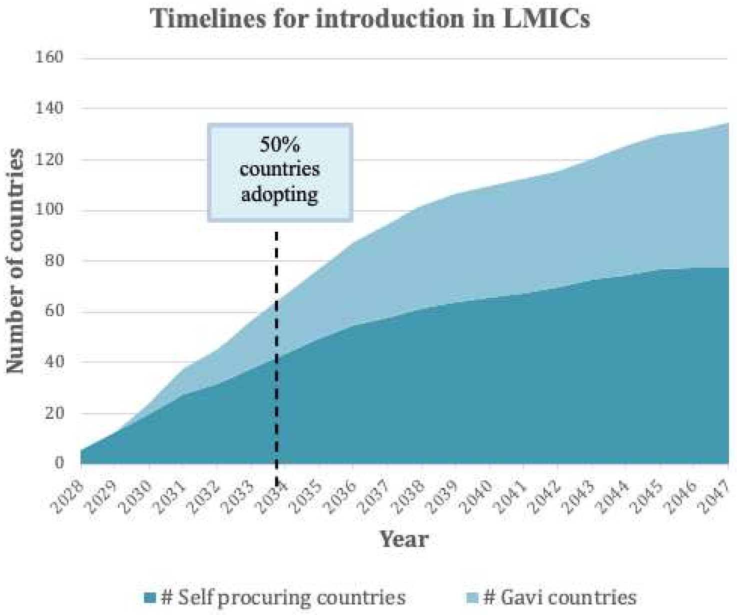

Figure L. Assumed cumulative number of countries introducing a novel vaccine per year.

Portnoy A, *et al. BMJ Glob Health* 2023; 8:e012466. doi: 10.1136/bmjgh-2023-012466

S1.8. Vaccine coverage targets.

For each vaccine implementation scenario, low, medium, and high coverage targets for 5 years post-introduction were evaluated. The medium coverage target for the routine infant vaccination was 85%, based on the 2019 DTP3 (diphtheria, tetanus toxoid, and pertussis) average coverage level according to the WHO and UNICEF estimates of national immunisation coverage, with 10% uncertainty (low coverage = 75%, high coverage = 95%).[28] Routine adolescent vaccination assumed a medium coverage target of 80% aligning with HPV coverage in South Africa combined with aggregated secondary school enrolment in China and India as assumed in Harris 2020,[29] also with 10% uncertainty targets (low coverage = 70%, high coverage = 90%). The medium coverage target for the adolescent/adult campaign was 70% aligning with the lower bound of the MenAfriVac campaigns in sub-Saharan Africa as assumed in Harris 2020,[29] with a wider uncertainty of 20% (low coverage = 50%, high coverage = 90%).

In the *Accelerated Scale-up* implementation, the 5-year coverage targets were achieved instantly in year 1, while in the *Basecase* and *Routine Only* implementations, the scale-up to coverage occurred linearly over 5 years.

S1.9. Calibrated *No-New-Vaccine* baseline trends.

Here we show the tuberculosis incidence rates plotted from 2000–2050 for the selected grouping for reporting model outcomes. In Figure M, looking by WHO region, we see the incidence rates are highest in AFR and SEAR, and lowest in AMR and EUR. In Figure N, we show the incidence rate trends by income group. Both incidence and mortality rates follow a trend with the highest estimated medians in lower-middle-income countries, followed by low-income countries and high-income countries, which aligns with the expectation of burden within each region. In Figure O, we compare incidence rates between countries included on the WHO high TB burden list and all other countries modelled, and as expected, higher values are predicted for countries on the high TB burden list.

Figure M. Tuberculosis incidence rates for the *No-New-Vaccine* baseline by WHO region.

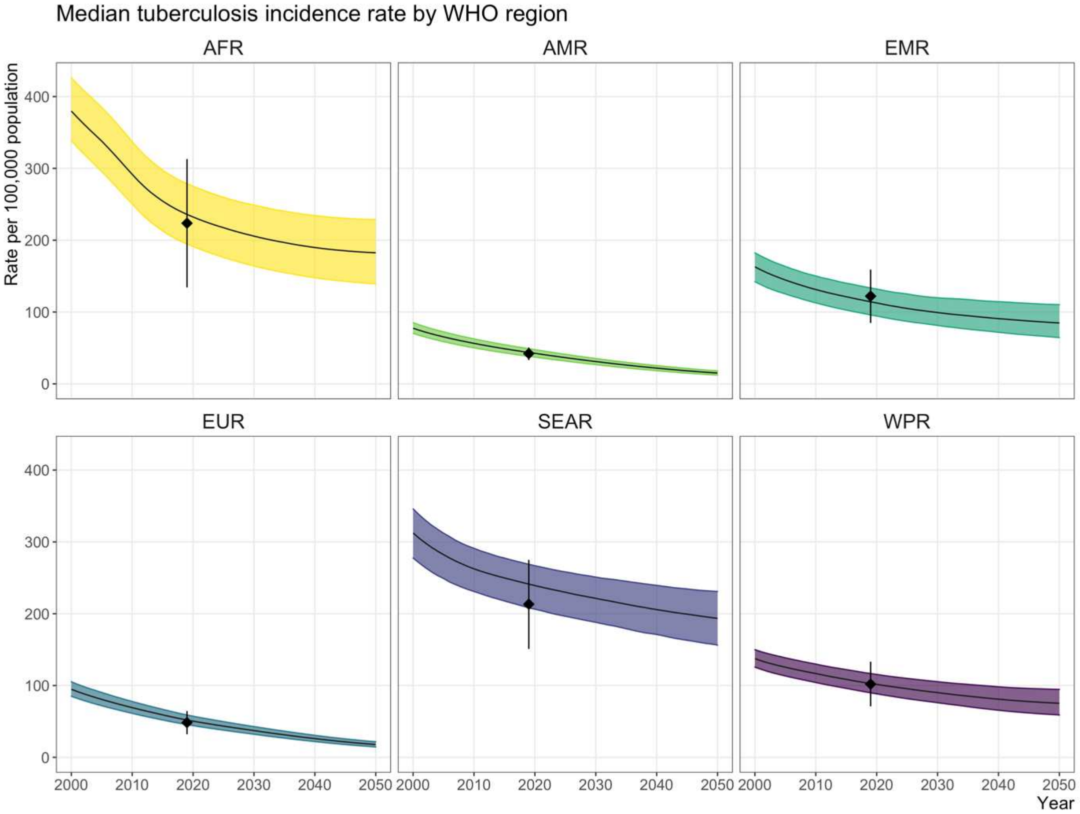

Note: The black diamond is the WHO median estimate of the incidence in 2019 for the 105 modelled LMICs by WHO region with 95% uncertainty range. The black line is the model estimated median incidence rate, with shaded 95% uncertainty ranges. AFR = WHO African Region, AMR = WHO Region of the Americas, EMR = WHO Eastern Mediterranean Region, EUR = WHO European Region, SEAR = WHO South-East Asian Region, WPR = WHO Western Pacific Region.

Figure N. Tuberculosis incidence rates for the *No-New-Vaccine* baseline by income group.

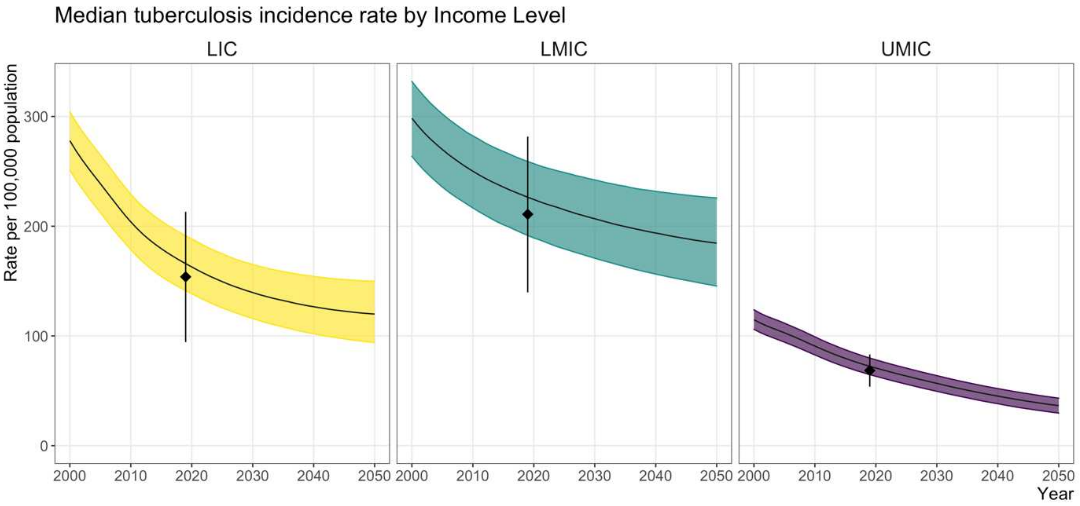

Note: The black diamond is the WHO median estimate of the incidence rate in 2019 for the 105 modelled LMICs by income group with 95% uncertainty range. The black line is the model estimated median incidence rate, with shaded 95% uncertainty ranges. LIC = low-income countries, LMIC = lower middle-income countries, UMIC = upper middle-income countries.

Figure O. Tuberculosis incidence rates for the *No-New-Vaccine* baseline for the countries included on the WHO high-TB-burden list and for all other countries modelled.

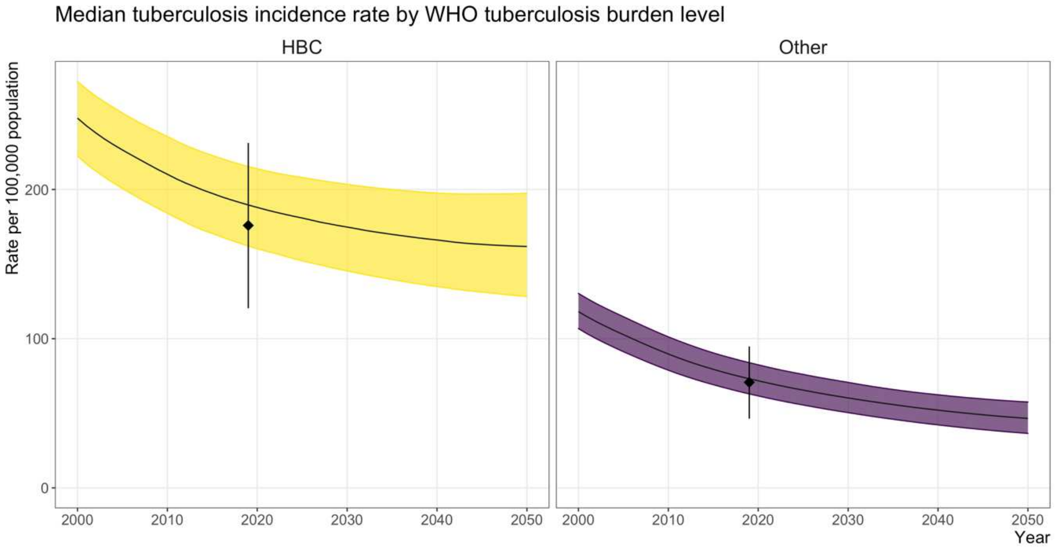

Note: The black diamond is the WHO median estimate of the incidence rate in 2019 for the 105 modelled LMICs by burden level with 95% uncertainty range. The black line is the model estimated median incidence rate, with shaded 95% uncertainty ranges. HBC = high burden countries.

S1.10. Model Parameters and Data Sources.

Parameters used in the natural history model structure and the HIV and ART model structure are provided in Table P below, along with their definitions, sources, and information on whether the parameter is fixed or varied (as well as whether they are varied by age or time) during calibration. The parameter ranges provided for the tuberculosis natural history parameters are priors fitted during calibration in a Bayesian analysis. We assume that all values within the prior range are equally likely. The prior ranges were pre-specified based on literature review and were reviewed as new data became available.

Portnoy A, *et al. BMJ Glob Health* 2023; 8:e012466. doi: 10.1136/bmjgh-2023-012466

Table P. Demographic and tuberculosis natural history parameters and definitions.

Description	Units	Symbol	Prior	Fixed or Varying During Calibration	Age Varying	Time Varying	Source
Births and deaths (excluding on-treatment mortality)							
Birth rate	Per year	B_k	United Nations World Population Prospects population estimates and projections	Fixed	No	Yes	[30]
Background mortality rate	Per year	$\mu_{j,k}$	Calculated in the model from United Nations population estimates and projections	Fixed	Yes, age specific mortality rates from demographic dataset	Yes	[30]
Mortality rate for clinical tuberculosis disease	Per person per year	μ_{DC_j}	(0–0·178)	Varying	Yes, value for children is greater than value for adults	No	[31]
Mortality rate post-tuberculosis disease	Per person per year	μ_{R_j}	$0.22\mu_{j,k}$	Fixed relationship	Yes, because $\mu_{j,k}$ varies	Yes, because $\mu_{j,k}$ varies	[32]
Natural history							
Force of infection	Per year	λ_j	Fitted	Fixed equation	Yes, age specific contact rates[9]	No	*Calculated*
Probability of transmission per infectious contact	-	p_T	(0–0·0068)	Varying	No	No	*Assumed*
Fraction of total tuberculosis disease that is extrapulmonary	-	ep	Country-specific average of previous 3 years	Fixed	No	No	[33, 34]

Infectiousness of subclinical relative to clinical tuberculosis	-	r	0·80	Fixed	No	No	[35]
Rate of self-clearance from I_F to U_C	Per person per year	ϕ_F	0·00000140	Fixed	No	No	[3]
Rate of self-clearance from I_S to U_C	Per person per year	ϕ_S	(0·0254–0·0467)	Varying	No	No	[3]
Rate of fast progression to disease, by age	Per person per year	θ_j	(0·0696–0·111)	Varying	Yes, value for children is less than value for adults	No	[3]
Rate from I_F to I_S	Per person per year	ω	0·5	Fixed	No	No	*Defined*
Rate of reactivation from I_S, by age	Per person per year	σ_j	(0·000135–0·00113)	Varying	Yes, value for children is less than value for adults	No	[3]
Rate of progression from D_S to D_C	Per person per year	ζ	(0–1)	Varying	No	No	*Assumed*
Rate of natural cure from D_C and D_S	Per person per year	χ	(0·1–0·25)	Varying	No	No	[36, 37]
Rate of relapse from R, by age	Per person per year	ρ_j	(0·0001–0·07)	Varying	Yes, value for children is less than value for adults	No	[38-40]
Treatment outcome parameters							
Treatment duration	Number of years	τ	0·5	Fixed	No	No	[4, 5]

Portnoy A, *et al. BMJ Glob Health* 2023; 8:e012466. doi: 10.1136/bmjgh-2023-012466

Rate of on-treatment mortality	Per person per year	$\mu_{T_j} = \frac{\kappa_j}{\tau}$	Country-specific	Varying	Yes, value for children greater than value for adults	Yes	[41]
Rate of treatment completion	Per person per year	$\frac{s_j}{\tau}$	Country-specific	Fixed equation	Yes, indirectly scaled by s_{Age}	Yes	[41]
Rate of treatment non-completion	Per person per year	$\frac{f_j}{\tau}$	Country-specific	Fixed equation	Yes, indirectly scaled by s_{Age}	Yes	[41]
Protection parameters							
Protection from reinfection for I_S, I_F, R	-	p_R	(0·6–0·85)	Varying	No	No	[7, 36, 37, 42, 43]
Relative protection from reinfection for self-clearance compared to p_R	-	p_C	0·50	Fixed	No	No	*Assumed*
SES parameter	-	p_E	(0–1)	Varying	No	No	*Assumed*
HIV parameters							
HIV incidence rate fitting factor	-	$\lambda_{H\text{fit}}$	(0–300)	Varying	No	No	*Fitted*
Rate of ART initiation fitting factor	-	$\alpha_{H\text{fit}}$	(0–7000)	Varying	No	No	*Fitted*
Rate of ART discontinuation	Per year	β_H	0·074	Fixed	No	No	[44, 45]
Mortality rate from HIV not on ART	Per year	μ_H	0·10	Fixed	No	No	[46]

Mortality rate from HIV on ART	Per year	δ_H	0·026	Fixed	No	No	[47]
Relative increase in progression rate for HIV_1	-	θ_{mul}	(3·94–14·45)	Varying	No	No	[48]
Relative reduction in θ_{mul} for HIV and ART compartments	-	$th1$	HIV_0 = 0<br>HIV_1 = 1·00<br>ART = 0·35	Fixed	No	No	[8]
Relative mortality rate adjustment for HIV and ART compartments	-	m_{adj}	HIV_0 = 1·00<br>HIV_1 = 1·50<br>ART = 1·15	Fixed	No	No	[8, 31, 49, 50]

Appendix S2. Analysed low- and middle-income country list.

Country	WHO Region	Income level[a]	Gavi status	High-TB burden[b]	High-TB/HIV burden[b]	High-MDR/RR-TB burden[b]	Base-case vaccine introduction year
Afghanistan	EMR	LIC	Gavi	No	No	No	2031
Angola	AFR	LMIC	Gavi	Yes	No	Yes	2032
Albania	EUR	UMIC	Non-Gavi	No	No	No	2035
Argentina	AMR	UMIC	Non-Gavi	No	No	No	2031
Armenia	EUR	UMIC	Gavi	No	No	No	2033
Azerbaijan	EUR	UMIC	Gavi	No	No	Yes	2028
Burundi	AFR	LIC	Gavi	No	No	No	2044
Benin	AFR	LMIC	Gavi	No	No	No	2037
Burkina Faso	AFR	LIC	Gavi	No	No	No	2039
Bangladesh	SEAR	LMIC	Gavi	Yes	No	Yes	2035
Bulgaria	EUR	UMIC	Non-Gavi	No	No	No	2029
Belarus	EUR	UMIC	Non-Gavi	No	No	Yes	2028
Bolivia	AMR	LMIC	Gavi	No	No	No	2037
Brazil	AMR	UMIC	Non-Gavi	Yes	Yes	No	2030
Bhutan	SEAR	LMIC	Gavi	No	No	No	2034
Botswana	AFR	UMIC	Non-Gavi	No	Yes	No	2028
Central African Republic	AFR	LIC	Gavi	Yes	Yes	No	2033
China	WPR	UMIC	Non-Gavi	Yes	No	Yes	2029
Côte d'Ivoire	AFR	LMIC	Gavi	No	No	No	2034

Cameroon	AFR	LMIC	Gavi	No	Yes	No	2031
Colombia	AMR	UMIC	Non-Gavi	No	No	No	2030
Costa Rica	AMR	UMIC	Non-Gavi	No	No	No	2033
Cuba	AMR	UMIC	Gavi	No	No	No	2035
Dominican Republic	AMR	UMIC	Non-Gavi	No	No	No	2031
Ecuador	AMR	UMIC	Non-Gavi	No	No	No	2033
Egypt	EMR	LMIC	Non-Gavi	No	No	No	2033
Eritrea	AFR	LIC	Gavi	No	No	No	2047
Ethiopia	AFR	LIC	Gavi	Yes	Yes	No	2030
Fiji	WPR	UMIC	Non-Gavi	No	No	No	2031
Gabon	AFR	UMIC	Non-Gavi	Yes	Yes	No	2038
Georgia	EUR	UMIC	Gavi	No	No	No	2029
Ghana	AFR	LMIC	Gavi	No	No	No	2040
Guinea	AFR	LIC	Gavi	No	Yes	No	2033
Gambia	AFR	LIC	Gavi	No	No	No	2039
Equatorial Guinea	AFR	UMIC	Non-Gavi	No	No	No	2042
Guatemala	AMR	UMIC	Non-Gavi	No	No	No	2036
Honduras	AMR	LMIC	Gavi	No	No	No	2037
Indonesia	SEAR	UMIC	Gavi	Yes	Yes	Yes	2034
India	SEAR	LMIC	Gavi	Yes	Yes	Yes	2033
Iran	EMR	UMIC	Non-Gavi	No	No	No	2031
Iraq	EMR	UMIC	Non-Gavi	No	No	No	2033

Portnoy A, *et al. BMJ Glob Health* 2023; 8:e012466. doi: 10.1136/bmjgh-2023-012466

Jordan	EMR	UMIC	Non-Gavi	No	No	No	2037
Kazakhstan	EUR	UMIC	Non-Gavi	No	No	Yes	2028
Kenya	AFR	LMIC	Gavi	Yes	Yes	No	2032
Kyrgyz Republic	EUR	LMIC	Gavi	No	No	Yes	2044
Cambodia	WPR	LMIC	Gavi	No	No	No	2036
Lao People’s Democratic Republic	WPR	LMIC	Gavi	No	No	No	2035
Liberia	AFR	LIC	Gavi	Yes	Yes	No	2037
Libya	EMR	UMIC	Non-Gavi	No	No	No	2035
Sri Lanka	SEAR	LMIC	Gavi	No	No	No	2028
Lesotho	AFR	LMIC	Gavi	Yes	Yes	No	2039
Morocco	EMR	LMIC	Non-Gavi	No	No	No	2029
Moldova, Republic of	EUR	LMIC	Gavi	No	No	Yes	2034
Madagascar	AFR	LIC	Gavi	No	No	No	2031
Maldives	SEAR	UMIC	Non-Gavi	No	No	No	2034
Mexico	AMR	UMIC	Non-Gavi	No	No	No	2029
Mali	AFR	LIC	Gavi	No	No	No	2037
Myanmar	SEAR	LMIC	Gavi	Yes	Yes	Yes	2031
Montenegro	EUR	UMIC	Non-Gavi	No	No	No	2044
Mongolia	WPR	LMIC	Gavi	Yes	No	Yes	2032
Mozambique	AFR	LIC	Gavi	Yes	Yes	Yes	2032
Mauritania	AFR	LMIC	Gavi	No	No	No	2042
Malawi	AFR	LIC	Gavi	No	Yes	No	2038

Malaysia	WPR	UMIC	Non-Gavi	No	No	No	2028
Namibia	AFR	UMIC	Non-Gavi	Yes	Yes	No	2030
Niger	AFR	LIC	Gavi	No	No	No	2036
Nigeria	AFR	LMIC	Gavi	Yes	Yes	Yes	2030
Nicaragua	AMR	LMIC	Gavi	No	No	No	2047
Nepal	SEAR	LMIC	Gavi	No	No	Yes	2036
Pakistan	EMR	LMIC	Gavi	Yes	No	Yes	2031
Peru	AMR	UMIC	Non-Gavi	No	No	Yes	2029
Philippines	WPR	LMIC	Non-Gavi	Yes	Yes	Yes	2030
Papua New Guinea	WPR	LMIC	Gavi	Yes	No	Yes	2032
Paraguay	AMR	UMIC	Non-Gavi	No	No	No	2035
Russian Federation	EUR	UMIC	Non-Gavi	No	Yes	Yes	2030
Rwanda	AFR	LIC	Gavi	No	No	No	2045
Sudan	EMR	LIC	Gavi	No	No	No	2036
Senegal	AFR	LMIC	Gavi	No	No	No	2038
Solomon Islands	WPR	LMIC	Gavi	No	No	No	2047
Sierra Leone	AFR	LIC	Gavi	Yes	No	No	2037
El Salvador	AMR	LMIC	Non-Gavi	No	No	No	2039
Serbia	EUR	UMIC	Non-Gavi	No	No	No	2036
South Sudan	AFR	LIC	Gavi	No	No	No	2034
São Tomé and Principe	AFR	LMIC	Gavi	No	No	No	2044
Suriname	AMR	UMIC	Non-Gavi	No	No	No	2040

Portnoy A, *et al. BMJ Glob Health* 2023; 8:e012466. doi: 10.1136/bmjgh-2023-012466

Swaziland	AFR	LMIC	Non-Gavi	No	Yes	No	2036
Syrian Arab Republic	EMR	LIC	Gavi	No	No	No	2036
Chad	AFR	LIC	Gavi	No	No	No	2033
Togo	AFR	LIC	Gavi	No	No	No	2041
Thailand	SEAR	UMIC	Non-Gavi	Yes	Yes	No	2031
Tajikistan	EUR	LIC	Gavi	No	No	Yes	2045
Timor-Leste	SEAR	LMIC	Gavi	No	No	No	2031
Tunisia	EMR	LMIC	Non-Gavi	No	No	No	2036
Turkey	EUR	UMIC	Non-Gavi	No	No	No	2030
Tanzania, United Republic of	AFR	LMIC	Gavi	Yes	Yes	No	2031
Uganda	AFR	LIC	Gavi	Yes	Yes	No	2034
Ukraine	EUR	LMIC	Non-Gavi	No	No	Yes	2033
Uzbekistan	EUR	LMIC	Gavi	No	No	Yes	2038
Venezuela	AMR	UMIC	Non-Gavi	No	No	No	2035
Vietnam	WPR	LMIC	Gavi	Yes	No	Yes	2038
Vanuatu	WPR	LMIC	Non-Gavi	No	No	No	2042
Yemen	EMR	LIC	Gavi	No	No	No	2036
South Africa	AFR	UMIC	Non-Gavi	Yes	Yes	Yes	2029
Zambia	AFR	LIC	Gavi	Yes	Yes	Yes	2034
Zimbabwe	AFR	LMIC	Gavi	No	Yes	Yes	2032

[a] LIC: Gross national income (GNI) per capita of $1,085 or less; LMIC: GNI per capita of $1,086 to $4,225; UMIC: GNI per capita of $4,256 to $13,205 (World Bank 2021).
[b] High-TB, high-TB/HIV (HIV-associated TB), and high-MDR/RR-TB (multidrug/rifampicin-resistant TB) burden countries as defined by the World Health Organization.[51]

Note: Vaccine introduction was assumed to commence in 2028 and end in 2047. See Clark et al. (https://doi.org/10.1016/S2214-109X(23)00045-1)[1] for introduction year methodology. AFR = African region; AMR = Region of the Americas; EMR = Eastern Mediterranean region; EUR = European region; LIC = low-income; LMIC = lower middle-income; SEAR = Southeast Asian region; TB = tuberculosis; UMIC = upper middle-income; WPR = Western Pacific region.

Portnoy A, *et al. BMJ Glob Health* 2023; 8:e012466. doi: 10.1136/bmjgh-2023-012466

Appendix S3. Sources and values of analytic inputs

Input	Value/Assumption/Formula	Source
**Total costs borne by TB-affected households, per episode**	Stratified by country, income quintile, and cost category	[52]
**Fraction of TB cases incurring catastrophic costs**	Stratified by country and income quintile	[52]
**Risk ratio of TB disease across income strata**	Poorest quintile: Reference<br>Poorer quintile: 0.8479<br>Middle quintile: 0.7477<br>Richer quintile: 0.6221<br>Richest quintile: 0.5157	Quantitative synthesis of TB prevalence surveys[9-19]
**Total costs borne by TB-affected households that are not accessing care/ with untreated TB, compared to those accessing treatment**	100% (50% and 150% in sensitivity analyses)	Assumption
**Discount rate**	Health outcomes: 0%<br>Cost outcomes: 0% (3% in sensitivity analysis)	[53]

Note: TB = tuberculosis.

Appendix S4. Costs borne by TB-affected households averted by infant tuberculosis vaccines (in millions), costs discounted at 3%.

Country grouping	Direct medical costs borne by TB-affected households averted	Direct non-medical costs borne by TB-affected households averted	Indirect costs borne by TB-affected households averted	Total costs borne by TB-affected households averted
All countries	629 (560–693)	1371 (1228–1516)	1584 (1429–1757)	3584 (3241–3953)
High-TB burden[a]	495 (431–554)	1206 (1069–1353)	1284 (1141–1442)	2986 (2654–3332)
High-TB/HIV burden[a]	405 (343–461)	1045 (910–1187)	1123 (987–1273)	2573 (2246–2910)
High-MDR/RR-TB burden[a]	477 (414–535)	1081 (948–1223)	1165 (1021–1322)	2722 (2399–3068)
Income level[b]				
LIC	109 (87.4–132)	190 (163–221)	360 (293–434)	659 (544–780)
LMIC	422 (362–482)	1045 (911–1191)	940 (822.8–1073)	2407 (2101–2748)
UMIC	98.0 (84.4–112)	136 (110–164)	284 (210–372)	518 (410–644)
World region				
AFR	293 (242–338)	654 (559–744)	754 (644–866)	1701 (1463–1929)
AMR	16.9 (14.6–19.3)	16.2 (14.5–18.0)	17.6 (15.7–19.7)	50.7 (45.5–56.1)
EMR	115 (90.2–142)	184 (141–230)	311 (238–381)	611 (474–752)
EUR	13.2 (10.7–16.0)	6.46 (5.53–7.37)	9.03 (7.55–10.5)	28.7 (23.8–33.6)
SEAR	114 (86.3–149)	376 (292–476)	307 (242–390)	797 (616–1018)
WPR	76.3 (64.3–89.8)	134 (107–162)	185 (143–230)	395 (317–477)

Note: Values in parentheses represent equal-tailed 95% credible intervals. Total costs included patient direct medical, direct non-medical, and indirect costs (all undiscounted) in 2020 USD.

[a] High-TB, high-TB/HIV (HIV-associated TB), and high-MDR/RR-TB (multidrug/rifampicin-resistant TB) burden countries as defined by the World Health Organization.

[b] LIC: Gross national income (GNI) per capita of $1,085 or less; LMIC: GNI per capita of $1,086 to $4,225; UMIC: GNI per capita of $4,256 to $13,205 (World Bank 2021).

Note: All countries include 105 low- and middle-income countries analysed. AFR = African region; AMR = Region of the Americas; EMR = Eastern Mediterranean region; EUR = European region; GDP = gross domestic product; LIC = low-income; LMIC = lower middle-income; SEAR = Southeast Asian region; UMIC = upper middle-income; WPR = Western Pacific region.

Appendix S5. Costs borne by TB-affected households averted by adolescent/adult tuberculosis vaccines (in millions), costs discounted at 3%.

Country grouping	Direct medical costs borne by TB-affected households averted	Direct non-medical costs borne by TB-affected households averted	Indirect costs borne by TB-affected households averted	Total costs borne by TB-affected households averted
All countries	4963 (4625–5302)	10110 (9471–10810)	11279 (10563–12109)	26352 (24817–28081)
High-TB burden[a]	3798 (3496–4093)	8700 (8062–9359)	9332 (8659–10084)	21831 (20374–23449)
High-TB/HIV burden[a]	2558 (2326–2806)	7179 (6568–7825)	7789 (7130–8517)	17526 (16096–19046)
High-MDR/RR-TB burden[a]	3864 (3564–4190)	7892 (7271–8533)	8598 (7923–9334)	20354 (18855–21937)
Income level[b]				
LIC	574 (504–650)	1103 (1005–1208)	1860 (1640–2101)	3537 (3154–3934)
LMIC	2742 (2484–3008)	7044 (6421–7672)	6280 (5743–6848)	16067 (14699–17426)
UMIC	1647 (1478–1829)	1962 (1813–2122)	3139 (2748–3547)	6748 (6152–7345)
World region				
AFR	1415 (1286–1560)	3393 (3122–3678)	4461 (4032–4928)	9268 (8511–10094)
AMR	298 (267–332)	385 (355–415)	372 (342–404)	1056 (978–1133)
EMR	567 (482–660)	950 (793–1110)	1448 (1212–1704)	2965 (2516–3453)
EUR	292 (248–343)	155 (139–171)	208 (182–235)	655 (577–741)
SEAR	1116 (936–1314)	3714 (3192–4282)	2879 (2449–3335)	7709 (6586–8901)
WPR	1275 (1128–1432)	1513 (1372–1666)	1911 (1692–2154)	4699 (4249–5192)

Note: Values in parentheses represent equal-tailed 95% credible intervals. Total costs included patient direct medical, direct non-medical, and indirect costs (all undiscounted) in 2020 USD.

[a] High-TB, high-TB/HIV (HIV-associated TB), and high-MDR/RR-TB (multidrug/rifampicin-resistant TB) burden countries as defined by the World Health Organization.

[b] LIC: Gross national income (GNI) per capita of $1,085 or less; LMIC: GNI per capita of $1,086 to $4,225; UMIC: GNI per capita of $4,256 to $13,205 (World Bank 2021).

Note: All countries include 105 low- and middle-income countries analysed. AFR = African region; AMR = Region of the Americas; EMR = Eastern Mediterranean region; EUR = European region; GDP = gross domestic product; LIC = low-income; LMIC = lower middle-income; SEAR = Southeast Asian region; UMIC = upper middle-income; WPR = Western Pacific region.

Portnoy A, *et al. BMJ Glob Health* 2023; 8:e012466. doi: 10.1136/bmjgh-2023-012466

Appendix S6. Costs borne by TB-affected households (in billions) and number of households with catastrophic costs (in millions) in the no-new-vaccine baseline.

Country grouping	Direct medical costs borne by TB-affected households	Direct non-medical costs borne by TB-affected households	Indirect costs borne by TB-affected households	Total costs borne by TB-affected households	Number of households with catastrophic costs
All countries	39.5 (36.7–42.4)	78.3 (73.5–83.3)	85.2 (79.3–91.3)	203 (191–215)	118 (111–126)
High-TB burden[a]	29.1 (26.7–31.5)	65.7 (60.8–70.8)	69.6 (64.3–75.4)	164 (153–176)	102 (94.8–110)
High-TB/HIV burden[a]	18.7 (16.8–20.7)	53.4 (48.7–58.4)	57.1 (52.0–62.4)	129 (118–141)	86.2 (78.9–93.9)
High-MDR/RR-TB burden[a]	30.6 (28.0–33.3)	60.1 (55.3–65.1)	64.3 (59.0–70.0)	155 (143–167)	91.5 (84.1–99.4)
Income level[b]					
LIC	4.27 (3.89–4.71)	8.46 (7.85–9.08)	13.3 (12.1–14.6)	26.1 (24.0–28.2)	16.7 (15.5–17.9)
LMIC	22.1 (20.0–24.2)	54.2 (49.4–58.9)	48.2 (43.9–52.3)	125 (114–135)	88.6 (80.9–95.9)
UMIC	13.1 (11.7–14.5)	15.6 (14.4–16.9)	23.6 (20.9–26.9)	52.4 (47.5–57.3)	12.8 (11.5–14.4)
World region					
AFR	10.1 (8.80–11.3)	24.2 (21.7–26.7)	32.3 (28.7–36.2)	66.6 (59.7–73.7)	40.9 (37.2–44.7)
AMR	2.51 (2.26–2.78)	3.49 (3.22–3.74)	3.24 (2.99–3.51)	9.24 (8.62–9.91)	2.02 (1.88–2.15)
EMR	3.75 (3.27–4.28)	6.48 (5.54–7.47)	9.10 (7.76–10.4)	19.3 (16.8–21.9)	10.8 (9.05–12.8)
EUR	3.29 (2.86–3.83)	1.59 (1.45–1.75)	2.25 (2.00–2.53)	7.14 (6.39–8.01)	1.16 (1.06–1.27)
SEAR	9.07 (7.73–10.4)	30.4 (26.7–34.4)	23.0 (19.9–26.2)	62.5 (54.6–70.9)	48.7 (42.7–55.1)
WPR	10.8 (9.41–12.2)	12.2 (10.9–13.5)	15.2 (13.5–17.3)	38.2 (34.4–42.5)	14.5 (12.5–16.8)

Note: Values in parentheses represent equal-tailed 95% credible intervals. Total costs included patient direct medical, direct non-medical, and indirect costs (all undiscounted) over 2028–2050 in 2020 USD. Catastrophic costs are defined as instances where the patient costs (either direct medical only or total) incurred during an episode of TB disease exceeded 20% of total annual household income.

[a] High-TB, high-TB/HIV (HIV-associated TB), and high-MDR/RR-TB (multidrug/rifampicin-resistant TB) burden countries as defined by the World Health Organization.

[b] LIC: Gross national income (GNI) per capita of $1,085 or less; LMIC: GNI per capita of $1,086 to $4,225; UMIC: GNI per capita of $4,256 to $13,205 (World Bank 2021).

Note: All countries include 105 low- and middle-income countries analysed. AFR = African region; AMR = Region of the Americas; EMR = Eastern Mediterranean region; EUR = European region; GDP = gross domestic product; LIC = low-income; LMIC = lower middle-income; SEAR = Southeast Asian region; UMIC = upper middle-income; WPR = Western Pacific region.

Appendix S7. Percent reduction in costs borne by TB-affected households averted and number of households with catastrophic costs averted by infant tuberculosis vaccines compared to no-new-vaccine baseline.

Country grouping	Direct medical costs borne by TB-affected households	Direct non-medical costs borne by TB-affected households	Indirect costs borne by TB-affected households	Total costs borne by TB-affected households	Number of households with catastrophic costs
All countries	2.62 (2.37–2.94)	2.89 (2.64–3.19)	3.06 (2.81–3.34)	2.91 (2.67–3.19)	3.13 (2.86–3.43)
High-TB burden[a]	2.80 (2.48–3.20)	3.03 (2.73–3.39)	3.03 (2.75–3.36)	2.99 (2.71–3.35)	3.24 (2.94–3.60)
High-TB/HIV burden[a]	3.58 (3.10–4.21)	3.24 (2.88–3.69)	3.24 (2.89–3.62)	3.29 (2.93–3.71)	3.30 (2.97–3.69)
High-MDR/RR-TB burden[a]	2.57 (2.26–2.95)	2.97 (2.65–3.36)	2.97 (2.67–3.32)	2.89 (2.59–3.25)	3.16 (2.83–3.56)
Income level[b]					
LIC	4.23 (3.62–4.94)	3.74 (3.34–4.24)	4.48 (3.88–5.24)	4.20 (3.66–4.86)	3.65 (3.26–4.20)
LMIC	3.17 (2.74–3.71)	3.19 (2.84–3.64)	3.23 (2.88–3.66)	3.20 (2.84–3.66)	3.22 (2.88–3.65)
UMIC	1.19 (1.07–1.34)	1.39 (1.16–1.67)	1.92 (1.52–2.44)	1.58 (1.30–1.93)	1.78 (1.41–2.25)
World region					
AFR	4.84 (4.04–5.94)	4.47 (3.86–5.31)	3.83 (3.34–4.42)	4.21 (3.65–4.95)	4.36 (3.82–5.06)
AMR	1.07 (0.99–1.16)	0.74 (0.68–0.81)	0.86 (0.79–0.94)	0.87 (0.81–0.95)	0.73 (0.66–0.80)
EMR	5.07 (4.23–6.01)	4.67 (3.83–5.67)	5.64 (4.68–6.74)	5.20 (4.34–6.16)	4.66 (3.77–5.79)
EUR	0.66 (0.58–0.76)	0.66 (0.59–0.73)	0.66 (0.58–0.74)	0.66 (0.58–0.74)	0.65 (0.58–0.74)
SEAR	2.10 (1.72–2.58)	2.07 (1.74–2.48)	2.23 (1.87–2.65)	2.13 (1.79–2.55)	2.08 (1.75–2.48)
WPR	1.13 (0.98–1.30)	1.78 (1.46–2.13)	1.97 (1.59–2.41)	1.67 (1.38–2.00)	2.59 (2.07–3.23)

Note: Values in parentheses represent equal-tailed 95% credible intervals. Total costs included patient direct medical, direct non-medical, and indirect costs (all undiscounted) over 2028–2050. Catastrophic costs are defined as instances where the patient costs (either direct medical only or total) incurred during an episode of TB disease exceeded 20% of total annual household income.

[a] High-TB, high-TB/HIV (HIV-associated TB), and high-MDR/RR-TB (multidrug/rifampicin-resistant TB) burden countries as defined by the World Health Organization.

[b] LIC: Gross national income (GNI) per capita of $1,085 or less; LMIC: GNI per capita of $1,086 to $4,225; UMIC: GNI per capita of $4,256 to $13,205 (World Bank 2021).

Note: All countries include 105 low- and middle-income countries analysed. AFR = African region; AMR = Region of the Americas; EMR = Eastern Mediterranean region; EUR = European region; GDP = gross domestic product; LIC = low-income; LMIC = lower middle-income; SEAR = Southeast Asian region; UMIC = upper middle-income; WPR = Western Pacific region.

Appendix S8. Percent reduction in costs borne by TB-affected households averted and number of households with catastrophic costs averted by adolescent/adult tuberculosis vaccines compared to no-new-vaccine baseline.

Country grouping	Direct medical costs borne by TB-affected households	Direct non-medical costs borne by TB-affected households	Indirect costs borne by TB-affected households	Total costs borne by TB-affected households	Number of households with catastrophic costs
All countries	2.62 (2.37–2.94)	2.89 (2.64–3.19)	3.06 (2.81–3.34)	2.91 (2.67–3.19)	3.13 (2.86–3.43)
High-TB burden[a]	2.80 (2.48–3.20)	3.03 (2.73–3.39)	3.03 (2.75–3.36)	2.99 (2.71–3.35)	3.24 (2.94–3.60)
High-TB/HIV burden[a]	3.58 (3.10–4.21)	3.24 (2.88–3.69)	3.24 (2.89–3.62)	3.29 (2.93–3.71)	3.30 (2.97–3.69)
High-MDR/RR-TB burden[a]	2.57 (2.26–2.95)	2.97 (2.65–3.36)	2.97 (2.67–3.32)	2.89 (2.59–3.25)	3.16 (2.83–3.56)
Income level[b]					
LIC	4.23 (3.62–4.94)	3.74 (3.34–4.24)	4.48 (3.88–5.24)	4.2 (3.66–4.86)	3.65 (3.26–4.20)
LMIC	3.17 (2.74–3.71)	3.19 (2.84–3.64)	3.23 (2.88–3.66)	3.2 (2.84–3.66)	3.22 (2.88–3.65)
UMIC	1.19 (1.07–1.34)	1.39 (1.16–1.67)	1.92 (1.52–2.44)	1.58 (1.3–1.93)	1.78 (1.41–2.25)
World region					
AFR	4.84 (4.04–5.94)	4.47 (3.86–5.31)	3.83 (3.34–4.42)	4.21 (3.65–4.95)	4.36 (3.82–5.06)
AMR	1.07 (0.99–1.16)	0.74 (0.68–0.81)	0.86 (0.79–0.94)	0.87 (0.81–0.95)	0.73 (0.66–0.80)
EMR	5.07 (4.23–6.01)	4.67 (3.83–5.67)	5.64 (4.68–6.74)	5.20 (4.34–6.16)	4.66 (3.77–5.79)
EUR	0.66 (0.58–0.76)	0.66 (0.59–0.73)	0.66 (0.58–0.74)	0.66 (0.58–0.74)	0.65 (0.58–0.74)
SEAR	2.10 (1.72–2.58)	2.07 (1.74–2.48)	2.23 (1.87–2.65)	2.13 (1.79–2.55)	2.08 (1.75–2.48)
WPR	1.13 (0.98–1.30)	1.78 (1.46–2.13)	1.97 (1.59–2.41)	1.67 (1.38–2.00)	2.59 (2.07–3.23)

Note: Values in parentheses represent equal-tailed 95% credible intervals. Total costs included patient direct medical, direct non-medical, and indirect costs (all undiscounted) over 2028–2050. Catastrophic costs are defined as instances where the patient costs (either direct medical only or total) incurred during an episode of TB disease exceeded 20% of total annual household income.

[a] High-TB, high-TB/HIV (HIV-associated TB), and high-MDR/RR-TB (multidrug/rifampicin-resistant TB) burden countries as defined by the World Health Organization.

[b] LIC: Gross national income (GNI) per capita of $1,085 or less; LMIC: GNI per capita of $1,086 to $4,225; UMIC: GNI per capita of $4,256 to $13,205 (World Bank 2021).

Note: All countries include 105 low- and middle-income countries analysed. AFR = African region; AMR = Region of the Americas; EMR = Eastern Mediterranean region; EUR = European region; GDP = gross domestic product; LIC = low-income; LMIC = lower middle-income; SEAR = Southeast Asian region; UMIC = upper middle-income; WPR = Western Pacific region.

Appendix S9. Tuberculosis cases averted, costs borne by TB-affected households averted, and number of households with catastrophic costs averted by infant and adolescent/adult tuberculosis vaccines (in millions) across 105 low- and middle-income countries by income quintile.

		Poorest	Poorer	Middle	Richer	Richest
Tuberculosis cases averted	Infant vaccine	2.06 (1.83–2.30)	1.74 (1.55–1.95)	1.18 (1.05–1.32)	0.98 (0.87–1.10)	0.81 (0.72–0.91)
	Adolescent/adult vaccine	9.55 (8.79–10.3)	8.1 (7.46–8.74)	5.6 (5.15–6.09)	4.66 (4.28–5.07)	3.86 (3.55–4.2)
Direct medical costs borne by TB-affected households averted	Infant vaccine	207 (182–230)	202 (179–225)	181 (160–200)	213 (189–235)	237 (210–265)
	Adolescent/adult vaccine	999 (924–1082)	984 (912–1059)	904 (837–976)	1076 (994–1159)	1213 (1110–1319)
Direct non-medical costs borne by TB-affected households averted	Infant vaccine	555 (495–616)	545 (483–605)	395 (354–440)	393 (352–439)	386 (346–431)
	Adolescent/adult vaccine	2589 (2402–2784)	2544 (2362–2735)	1895 (1756–2043)	1888 (1749–2035)	1854 (1710–2008)
Indirect costs borne by TB-affected households averted	Infant vaccine	246 (221–271)	440 (393–490)	470 (423–524)	663 (596–738)	799 (712–896)
	Adolescent/adult vaccine	1107 (1031–1185)	1974 (1842–2123)	2169 (2019–2343)	3049 (2838–3287)	3673 (3387–3988)
Total costs borne by TB-affected households averted	Infant vaccine	1007 (900–1115)	1187 (1058–1313)	1046 (942–1157)	1268 (1144–1401)	1423 (1274–1579)
	Adolescent/adult vaccine	4695 (4384–5019)	5502 (5151–5874)	4968 (4641–5339)	6014 (5637–6447)	6740 (6288–7231)
Number of households with catastrophic costs averted	Infant vaccine	1.47 (1.31–1.63)	0.97 (0.86–1.08)	0.55 (0.49–0.61)	0.41 (0.37–0.45)	0.32 (0.28–0.35)
	Adolescent/adult vaccine	6.62 (6.13–7.11)	4.26 (3.96–4.58)	2.44 (2.26–2.64)	1.81 (1.68–1.96)	1.41 (1.31–1.52)

Note: Total costs included patient direct medical, direct non-medical, and indirect costs (all undiscounted) in 2020 USD. Costs borne by TB-affected households are categorized as "catastrophic" if they exceed 20% of total household's annual income.

Appendix S10. Percentage of households experiencing catastrophic costs by gross domestic product (GDP) per capita and by income quintile in 105 low- and middle-income countries.

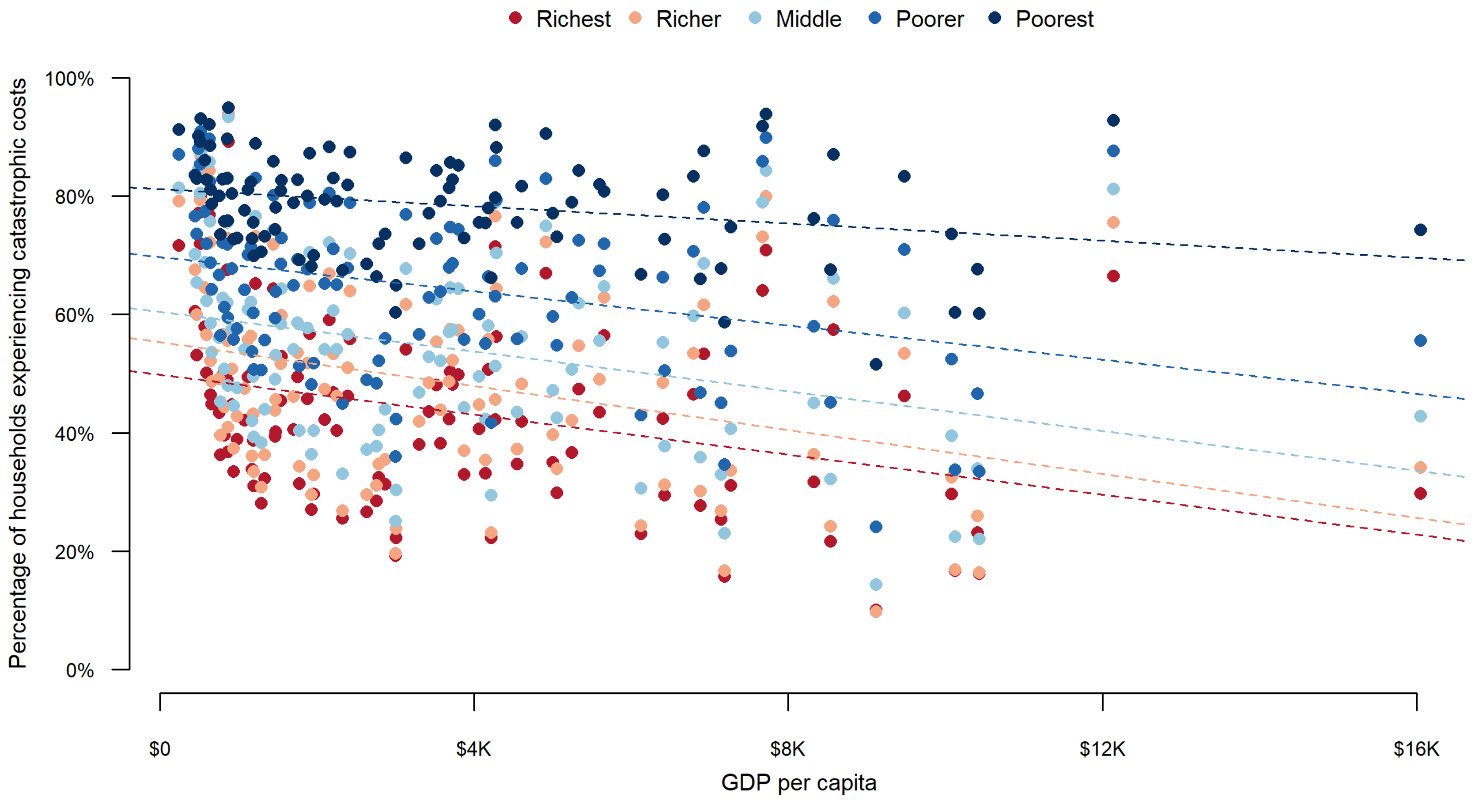

Appendix S11. Distribution of costs borne by TB-affected households averted by an adolescent/adult vaccine across all modelled strata, ordered by household income.

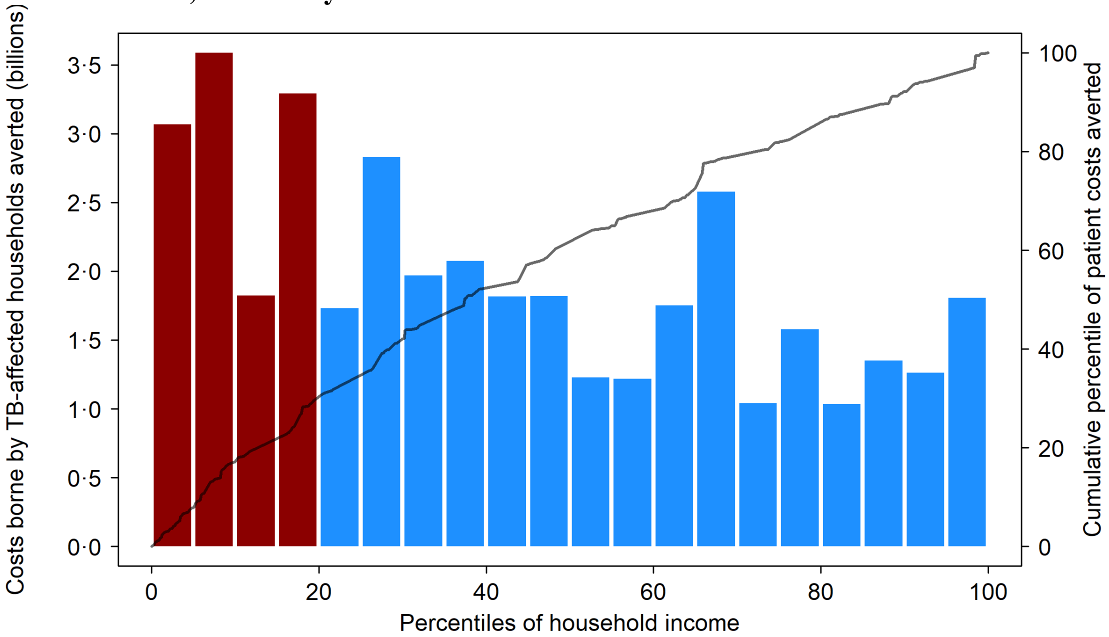

Note: TB = tuberculosis, CC = catastrophic costs. Ordering of population by household income based on average 2020 per capita GDP in purchasing power parity (PPP) dollars, for each modelled stratum (505 total strata). Bars shaded red indicate poorest 20% of modelled population by PPP GDP per capita.

Appendix S12. Costs borne by TB-affected households averted and number of households with catastrophic costs averted by adolescent/adult vaccines (in millions): 75% efficacy scenario.

Country grouping	Direct medical costs borne by TB-affected households averted	Direct non-medical costs borne by TB-affected households averted	Indirect costs borne by TB-affected households averted	Total costs borne by TB-affected households averted	Number of households with catastrophic costs averted
All countries	10613 (9889–11347)	21893 (20494–23438)	24305 (22742–26094)	56812 (53484–60618)	33.4 (31.2–35.8)
High-TB burden[a]	8094 (7456–8735)	18805 (17395–20295)	20086 (18629–21731)	46985 (43777–50586)	29.6 (27.3–31.8)
High-TB/HIV burden[a]	5546 (5040–6085)	15625 (14254–17071)	16861 (15409–18467)	38032 (34936–41398)	25.2 (23.1–27.4)
High-MDR/RR-TB burden[a]	8229 (7573–8945)	17047 (15674–18476)	18455 (16975–20092)	43732 (40469–47176)	26.1 (23.9–28.3)
Income level[b]					
LIC	1261 (1111–1427)	2443 (2227–2669)	4089 (3608–4616)	7793 (6961–8654)	4.82 (4.39–5.25)
LMIC	6023 (5454–6605)	15437 (14085–16812)	13720 (12541–14966)	35180 (32139–38183)	25.2 (23.0–27.3)
UMIC	3329 (2975–3690)	4014 (3694–4340)	6496 (5671–7359)	13839 (12574–15100)	3.40 (3.04–3.79)
World region					
AFR	3098 (2812–3417)	7429 (6833–8059)	9720 (8808–10731)	20246 (18603–22045)	12.5 (11.6–13.4)
AMR	610 (546–679)	792 (731–853)	762 (699–828)	2164 (2001–2327)	0.45 (0.42–0.48)
EMR	1212 (1026–1413)	2029 (1693–2384)	3080 (2574–3632)	6320 (5351–7363)	3.39 (2.78–4.06)
EUR	631 (536–746)	326 (291–361)	443 (388–502)	1400 (1231–1592)	0.22 (0.20–0.25)
SEAR	2454 (2061–2892)	8189 (7032–9432)	6323 (5374–7332)	16966 (14500–19571)	13.0 (11.2–14.9)
WPR	2609 (2304–2935)	3129 (2830–3465)	3978 (3509–4510)	9715 (8756–10750)	3.90 (3.38–4.52)

Note: Values in parentheses represent equal-tailed 95% credible intervals. Total costs included patient direct medical, direct non-medical, and indirect costs (all undiscounted) in 2020 USD. Costs borne by TB-affected households are categorized as “catastrophic” if they exceed 20% of total household’s annual income.
[a] High-TB, high-TB/HIV (HIV-associated TB), and high-MDR/RR-TB (multidrug/rifampicin-resistant TB) burden countries as defined by the World Health Organization.
[b] LIC: Gross national income (GNI) per capita of $1,085 or less; LMIC: GNI per capita of $1,086 to $4,225; UMIC: GNI per capita of $4,256 to $13,205 (World Bank 2021).
Note: All countries include 105 low- and middle-income countries analysed. AFR = African region; AMR = Region of the Americas; EMR = Eastern Mediterranean region; EUR = European region; GDP = gross domestic product; LIC = low-income; LMIC = lower middle-income; SEAR = Southeast Asian region; UMIC = upper middle-income; WPR = Western Pacific region.

Appendix S13. Costs borne by TB-affected households averted and number of households with catastrophic costs averted by infant vaccines (in millions): accelerated scale-up scenario.

Country grouping	Direct medical costs borne by TB-affected households averted	Direct non-medical costs borne by TB-affected households averted	Indirect costs borne by TB-affected households averted	Total costs borne by TB-affected households averted	Number of households with catastrophic costs averted
All countries	2441 (2190–2700)	5434 (4883–6053)	5972 (5397–6629)	13847 (12506–15274)	8.83 (7.93–9.80)
High-TB burden[a]	1800 (1571–2035)	4639 (4098–5253)	4717 (4213–5285)	11156 (9922–12518)	7.73 (6.87–8.70)
High-TB/HIV burden[a]	1484 (1260–1708)	4059 (3544–4647)	4123 (3637–4678)	9666 (8494–11002)	6.66 (5.84–7.58)
High-MDR/RR-TB burden[a]	1794 (1561–2036)	4179 (3655–4783)	4241 (3751–4803)	10214 (8997–11559)	6.77 (5.95–7.72)
Income level[b]					
LIC	449 (372–532)	807 (704–920)	1456 (1228–1721)	2712 (2308–3148)	1.54 (1.34–1.79)
LMIC	1686 (1461–1928)	4223 (3693–4836)	3708 (3263–4252)	9616 (8443–11011)	6.87 (6.04–7.85)
UMIC	306 (267–348)	405 (337–478)	808 (612–1042)	1519 (1229–1850)	0.42 (0.32–0.52)
World region					
AFR	1016 (862–1159)	2290 (2001–2581)	2614 (2297–2951)	5919 (5199–6649)	3.81 (3.37–4.28)
AMR	64.2 (55.8–72.5)	65.7 (59.8–73.1)	67.6 (60.8–75.3)	198 (179–218)	0.039 (0.035–0.044)
EMR	404 (316–492)	636 (494–791)	1071 (820–1313)	2111 (1649–2578)	1.06 (0.80–1.35)
EUR	122 (95.0–156)	46.3 (37.5–57.4)	75.7 (60.8–94.6)	243 (196–307)	0.04 (0.03–0.05)
SEAR	575 (430–755)	1955 (1536–2477)	1531 (1195–1942)	4061 (3173–5163)	3.12 (2.47–3.90)
WPR	261 (219–310)	441 (361–527)	612 (484–747)	1314 (1075–1558)	0.76 (0.59–0.93)

Note: Values in parentheses represent equal-tailed 95% credible intervals. Total costs included patient direct medical, direct non-medical, and indirect costs (all undiscounted) in 2020 USD. Costs borne by TB-affected households are categorized as "catastrophic" if they exceed 20% of total household's annual income.

[a] High-TB, high-TB/HIV (HIV-associated TB), and high-MDR/RR-TB (multidrug/rifampicin-resistant TB) burden countries as defined by the World Health Organization.

[b] LIC: Gross national income (GNI) per capita of $1,085 or less; LMIC: GNI per capita of $1,086 to $4,225; UMIC: GNI per capita of $4,256 to $13,205 (World Bank 2021).

Note: All countries include 105 low- and middle-income countries analysed. AFR = African region; AMR = Region of the Americas; EMR = Eastern Mediterranean region; EUR = European region; GDP = gross domestic product; LIC = low-income; LMIC = lower middle-income; SEAR = Southeast Asian region; UMIC = upper middle-income; WPR = Western Pacific region.

Appendix S14. Costs borne by TB-affected households averted and number of households with catastrophic costs averted by adolescent/adult tuberculosis vaccines (in millions): accelerated scale-up scenario.

Country grouping	Direct medical costs borne by TB-affected households averted	Direct non-medical costs borne by TB-affected households averted	Indirect costs borne by TB-affected households averted	Total costs borne by TB-affected households averted	Number of households with catastrophic costs averted
All countries	11021 (10308–11771)	22358 (20909–23896)	24279 (22813–25976)	57658 (54410–61314)	33.9 (31.7–36.3)
High-TB burden[a]	8015 (7389–8634)	18794 (17329–20281)	19566 (18171–21086)	46375 (43173–49852)	29.5 (27.2–31.8)
High-TB/HIV burden[a]	5505 (4986–6025)	15672 (14328–17104)	16345 (15020–17807)	37522 (34506–40766)	25.1 (23–27.4)
High-MDR/RR-TB burden[a]	8430 (7776–9130)	17210 (15762–18693)	18068 (16691–19547)	43708 (40371–47120)	26.3 (24.0–28.6)
Income level[b]					
LIC	1352 (1197–1524)	2579 (2369–2809)	4263 (3790–4788)	8194 (7370–9073)	4.97 (4.56–5.38)
LMIC	6537 (5976–7124)	16118 (14739–17531)	14291 (13060–15559)	36947 (33837–40094)	26.0 (23.8–28.2)
UMIC	3132 (2825–3482)	3660 (3401–3943)	5725 (5058–6420)	12517 (11464–13560)	3.00 (2.71–3.31)
World region					
AFR	3042 (2796–3296)	7198 (6706–7706)	9173 (8400–9995)	19413 (17988–20873)	12.0 (11.3–12.8)
AMR	648 (580–718)	825 (767–885)	794 (733–859)	2267 (2104–2425)	0.48 (0.44–0.51)
EMR	1197 (1020–1390)	1986 (1667–2305)	3013 (2535–3526)	6196 (5290–7196)	3.28 (2.71–3.89)
EUR	909 (774–1073)	415 (373–460)	609 (535–689)	1933 (1713–2190)	0.31 (0.28–0.35)
SEAR	2669 (2238–3114)	8974 (7684–10314)	6888 (5852–7939)	18531 (15779–21289)	14.2 (12.3–16.2)
WPR	2556 (2268–2879)	2961 (2707–3249)	3801 (3386–4258)	9318 (8475–10274)	3.64 (3.22–4.17)

Note: Values in parentheses represent equal-tailed 95% credible intervals. Total costs included patient direct medical, direct non-medical, and indirect costs (all undiscounted) in 2020 USD. Costs borne by TB-affected households are categorized as “catastrophic” if they exceed 20% of total household’s annual income.

[a] High-TB, high-TB/HIV (HIV-associated TB), and high-MDR/RR-TB (multidrug/rifampicin-resistant TB) burden countries as defined by the World Health Organization.

[b] LIC: Gross national income (GNI) per capita of $1,025 or less; LMIC: GNI per capita of $1,026 to $3,995; UMIC: GNI per capita of $3,996 to $12,375 (World Bank 2019).

Note: All countries include 105 low- and middle-income countries analysed. AFR = African region; AMR = Region of the Americas; EMR = Eastern Mediterranean region; EUR = European region; GDP = gross domestic product; LIC = low-income; LMIC = lower middle-income; SEAR = Southeast Asian region; UMIC = upper middle-income; WPR = Western Pacific region.

Appendix S15. Costs borne by TB-affected households averted and number of households with catastrophic costs averted by infant tuberculosis vaccines (in millions), assuming costs for untreated cases are 0.5x treated cases.

Country grouping	Direct medical costs borne by TB-affected households averted	Direct non-medical costs borne by TB-affected households averted	Indirect costs borne by TB-affected households averted	Total costs borne by TB-affected households averted	Number of households with catastrophic costs averted
All countries	713 (641–781)	1570 (1422–1718)	1885 (1713–2073)	4168 (3798–4572)	2.80 (2.53–3.07)
High-TB burden[a]	546 (481–609)	1371 (1228–1522)	1504 (1345–1675)	3422 (3075–3784)	2.49 (2.22–2.76)
High-TB/HIV burden[a]	441 (379–501)	1174 (1037–1319)	1305 (1161–1467)	2920 (2589–3280)	2.12 (1.88–2.36)
High-MDR/RR-TB burden[a]	525 (460–588)	1223 (1080–1377)	1360 (1208–1531)	3108 (2770–3474)	2.16 (1.91–2.42)
Income level[b]					
LIC	136 (110–165)	234 (202–271)	459 (379–553)	829 (694–982)	0.51 (0.44–0.60)
LMIC	464 (401–527)	1180 (1038–1337)	1092 (966–1238)	2736 (2416–3096)	2.12 (1.87–2.38)
UMIC	113 (98–129)	156 (130–185)	335 (255–426)	603 (489–734)	0.18 (0.14–0.23)
World region					
AFR	300 (252–346)	688 (599–783)	835 (721–951)	1822 (1588–2062)	1.31 (1.15–1.48)
AMR	21.8 (19.0–24.7)	20.5 (18.5–22.5)	22.7 (20.4–25.1)	65 (58.6–71.4)	0.012 (0.011–0.013)
EMR	146 (115–178)	230 (181–285)	402 (316–490)	778 (618–948)	0.42 (0.32–0.54)
EUR	16.7 (13.8–20.1)	8.14 (7.13–9.16)	11.6 (9.90–13.4)	36.5 (31.2–42.1)	0.006 (0.005–0.007)
SEAR	141 (109–178)	463 (368–570)	386 (312–477)	990 (789–1222)	0.77 (0.62–0.94)
WPR	87.7 (74.6–102)	161 (130–192)	228 (179–279)	477 (385–572)	0.29 (0.22–0.36)

Note: Values in parentheses represent equal-tailed 95% credible intervals. Total costs included patient direct medical, direct non-medical, and indirect costs (all undiscounted) in 2020 USD. Costs borne by TB-affected households are categorized as “catastrophic” if they exceed 20% of total household’s annual income.

[a] High-TB, high-TB/HIV (HIV-associated TB), and high-MDR/RR-TB (multidrug/rifampicin-resistant TB) burden countries as defined by the World Health Organization.

[b] LIC: Gross national income (GNI) per capita of $1,085 or less; LMIC: GNI per capita of $1,086 to $4,225; UMIC: GNI per capita of $4,256 to $13,205 (World Bank 2021).

Note: All countries include 105 low- and middle-income countries analysed. AFR = African region; AMR = Region of the Americas; EMR = Eastern Mediterranean region; EUR = European region; GDP = gross domestic product; LIC = low-income; LMIC = lower middle-income; SEAR = Southeast Asian region; UMIC = upper middle-income; WPR = Western Pacific region.

Portnoy A, *et al. BMJ Glob Health* 2023; 8:e012466. doi: 10.1136/bmjgh-2023-012466

Appendix S16. Costs borne by TB-affected households averted and number of households with catastrophic costs averted by adolescent/adult tuberculosis vaccines (in millions), assuming costs for untreated cases are 0.5x treated cases.

Country grouping	Direct medical costs borne by TB-affected households averted	Direct non-medical costs borne by TB-affected households averted	Indirect costs borne by TB-affected households averted	Total costs borne by TB-affected households averted	Number of households with catastrophic costs averted
All countries	5727 (5360–6096)	11734 (11011–12476)	13356 (12584–14211)	30817 (29181–32592)	18.7 (17.6–20.0)
High-TB burden[a]	4313 (3988–4636)	10067 (9358–10777)	10957 (10241–11712)	25337 (23755–26997)	16.5 (15.3–17.7)
High-TB/HIV burden[a]	2894 (2644–3141)	8281 (7606–8967)	9118 (8426–9856)	20293 (18746–21929)	14.0 (12.9–15.1)
High-MDR/RR-TB burden[a]	4405 (4075–4750)	9119 (8416–9817)	10073 (9374–10810)	23598 (22050–25247)	14.5 (13.4–15.7)
Income level[b]					
LIC	705 (625–794)	1339 (1229–1458)	2323 (2064–2617)	4367 (3924–4826)	2.85 (2.60–3.09)
LMIC	3163 (2899–3427)	8197 (7537–8902)	7480 (6863–8106)	18841 (17370–20375)	14.0 (12.8–15.1)
UMIC	1859 (1677–2053)	2197 (2049–2349)	3554 (3172–3950)	7610 (6999–8190)	1.90 (1.71–2.09)
World region					
AFR	1496 (1383–1613)	3651 (3418–3893)	5000 (4602–5453)	10148 (9466–10864)	6.77 (6.34–7.21)
AMR	361 (324–402)	457 (424–491)	451 (416–489)	1269 (1179–1359)	0.27 (0.25–0.29)
EMR	687 (589–796)	1143 (969–1329)	1797 (1533–2096)	3626 (3114–4177)	2.02 (1.68–2.37)
EUR	363 (312–422)	187 (169–204)	258 (229–289)	807 (719–907)	0.13 (0.12–0.15)
SEAR	1371 (1165–1583)	4555 (3948–5185)	3595 (3080–4114)	9522 (8178–10892)	7.33 (6.36–8.32)
WPR	1450 (1291–1609)	1740 (1594–1893)	2255 (2021–2498)	5445 (4966–5939)	2.21 (1.95–2.49)

Note: Values in parentheses represent equal-tailed 95% credible intervals. Total costs included patient direct medical, direct non-medical, and indirect costs (all undiscounted) in 2020 USD. Costs borne by TB-affected households are categorized as "catastrophic" if they exceed 20% of total household's annual income.

[a] High-TB, high-TB/HIV (HIV-associated TB), and high-MDR/RR-TB (multidrug/rifampicin-resistant TB) burden countries as defined by the World Health Organization.

[b] LIC: Gross national income (GNI) per capita of $1,085 or less; LMIC: GNI per capita of $1,086 to $4,225; UMIC: GNI per capita of $4,256 to $13,205 (World Bank 2021).

Note: All countries include 105 low- and middle-income countries analysed. AFR = African region; AMR = Region of the Americas; EMR = Eastern Mediterranean region; EUR = European region; GDP = gross domestic product; LIC = low-income; LMIC = lower middle-income; SEAR = Southeast Asian region; UMIC = upper middle-income; WPR = Western Pacific region.

Appendix S17. Total costs borne by TB-affected households averted in the base-case (Panel A) and assuming costs for untreated cases are 0.5x treated cases (Panel B) by within-country income quintile comparing infant vaccine to adolescent/adult vaccine.

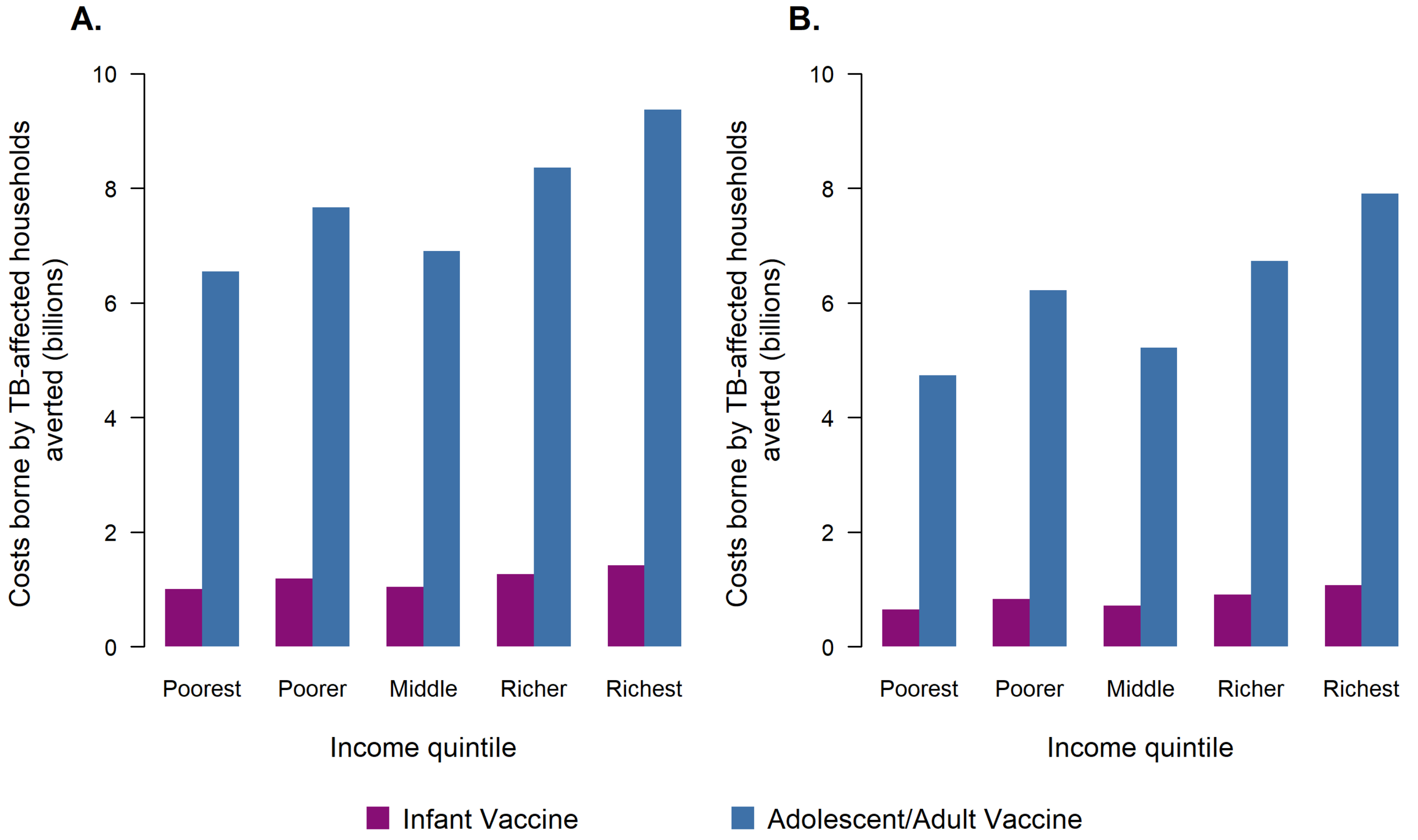

Note: Total costs included patient direct medical, direct non-medical, and indirect costs (all undiscounted) in 2020 USD.

Appendix S18. Number of households with catastrophic costs averted in the base-case (Panel A) and assuming costs for untreated cases are 0.5x treated cases (Panel B) by within-country income quintile comparing infant vaccine to adolescent/adult vaccine.

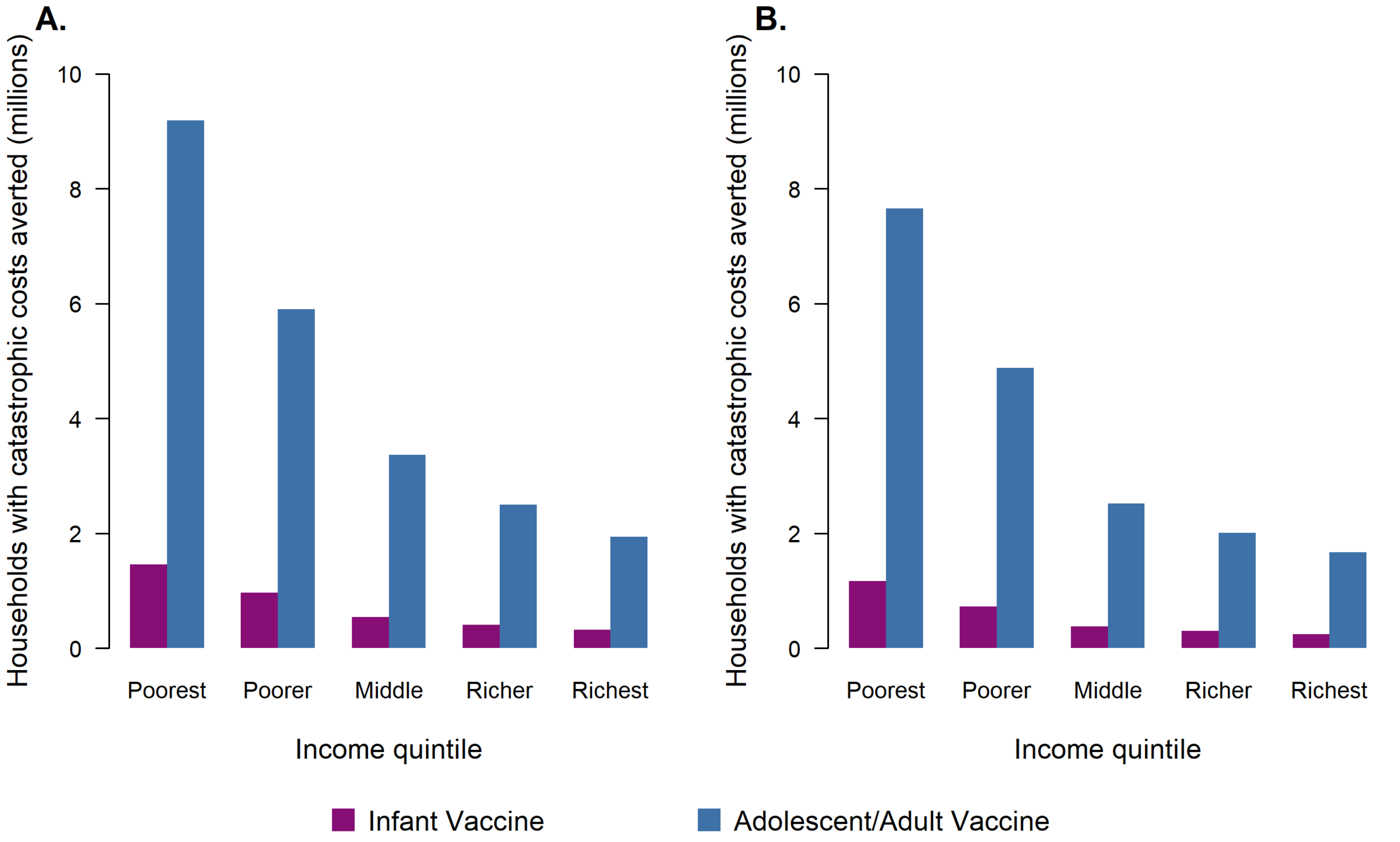

Note: Catastrophic costs included patient direct medical, direct non-medical, and indirect costs. Catastrophic costs are defined as instances where the patient costs incurred during an episode of TB disease exceed 20% of total annual household income.

Portnoy A, *et al. BMJ Glob Health* 2023; 8:e012466. doi: 10.1136/bmjgh-2023-012466

Appendix S19. Costs borne by TB-affected households averted and number of households with catastrophic costs averted by infant tuberculosis vaccines (in millions), assuming costs for untreated cases are 1.5x treated cases.

Country grouping	Direct medical costs borne by TB-affected households averted	Direct non-medical costs borne by TB-affected households averted	Indirect costs borne by TB-affected households averted	Total costs borne by TB-affected households averted	Number of households with catastrophic costs averted
All countries	1359 (1199–1504)	2958 (2629–3296)	3329 (2984–3712)	7646 (6855–8491)	4.21 (3.75–4.68)
High-TB burden[a]	1082 (934–1218)	2612 (2292–2949)	2720 (2391–3071)	6413 (5651–7216)	3.80 (3.35–4.27)
High-TB/HIV burden[a]	896 (752–1027)	2282 (1979–2607)	2391 (2081–2734)	5569 (4807–6346)	3.26 (2.85–3.70)
High-MDR/RR-TB burden[a]	1041 (895–1177)	2343 (2030–2678)	2465 (2147–2815)	5850 (5120–6649)	3.32 (2.89–3.77)
Income level[b]					
LIC	225 (180–274)	399 (337–467)	739 (596–895)	1364 (1119–1628)	0.66 (0.57–0.79)
LMIC	934 (794–1071)	2282 (1974–2615)	2016 (1752–2322)	5232 (4526–5995)	3.29 (2.87–3.76)
UMIC	199 (170–230)	278 (221–340)	574 (413–773)	1051 (813–1339)	0.26 (0.19–0.33)
World region					
AFR	667 (546–770)	1469 (1251–1674)	1641 (1391–1895)	3777 (3221–4300)	2.03 (1.77–2.30)
AMR	32.2 (27.7–37.0)	31.2 (27.5–35.0)	33.2 (29.4–37.5)	96.6 (85.9–108)	0.016 (0.014–0.018)
EMR	235 (179–291)	375 (282–472)	624 (466–776)	1234 (935–1530)	0.55 (0.40–0.72)
EUR	27.0 (21.4–33.3)	12.9 (10.8–15.0)	18.1 (14.8–21.7)	58.0 (47.3–69.7)	0.008 (0.007–0.010)
SEAR	241 (178–322)	798 (610–1028)	640 (497–831)	1679 (1286–2181)	1.17 (0.91–1.49)
WPR	157 (131–187)	272 (214–336)	372 (282–469)	802 (637–982)	0.43 (0.31–0.54)

Note: Values in parentheses represent equal-tailed 95% credible intervals. Total costs included patient direct medical, direct non-medical, and indirect costs (all undiscounted) in 2020 USD. Costs borne by TB-affected households are categorized as “catastrophic” if they exceed 20% of total household’s annual income.
[a] High-TB, high-TB/HIV (HIV-associated TB), and high-MDR/RR-TB (multidrug/rifampicin-resistant TB) burden countries as defined by the World Health Organization.
[b] LIC: Gross national income (GNI) per capita of $1,085 or less; LMIC: GNI per capita of $1,086 to $4,225; UMIC: GNI per capita of $4,256 to $13,205 (World Bank 2021).
Note: All countries include 105 low- and middle-income countries analysed. AFR = African region; AMR = Region of the Americas; EMR = Eastern Mediterranean region; EUR = European region; GDP = gross domestic product; LIC = low-income; LMIC = lower middle-income; SEAR = Southeast Asian region; UMIC = upper middle-income; WPR = Western Pacific region.

Appendix S20. Costs borne by TB-affected households averted and number of households with catastrophic costs averted by adolescent/adult tuberculosis vaccines (in millions), assuming costs for untreated cases are 1.5x treated cases.

Country grouping	Direct medical costs borne by TB-affected households averted	Direct non-medical costs borne by TB-affected households averted	Indirect costs borne by TB-affected households averted	Total costs borne by TB-affected households averted	Number of households with catastrophic costs averted
All countries	8777 (8141–9446)	18241 (16978–19648)	19883 (18497–21500)	46902 (43898–50346)	25.4 (23.6–27.2)
High-TB burden[a]	6734 (6155–7304)	15691 (14430–17061)	16445 (15154–17918)	38869 (35905–42123)	22.5 (20.7–24.3)
High-TB/HIV burden[a]	4720 (4260–5225)	13158 (11948–14530)	13896 (12627–15343)	31774 (28970–34823)	19.2 (17.5–21.0)
High-MDR/RR-TB burden[a]	6823 (6243–7448)	14232 (12967–15608)	15102 (13793–16555)	36158 (33232–39377)	19.9 (18.1–21.7)
Income level[b]					
LIC	1048 (913–1192)	2033 (1831–2243)	3362 (2924–3832)	6443 (5705–7218)	3.55 (3.22–3.89)
LMIC	5128 (4612–5664)	13059 (11839–14361)	11418 (10374–12493)	29605 (26902–32463)	19.3 (17.6–21.1)
UMIC	2601 (2323–2904)	3149 (2867–3444)	5104 (4373–5891)	10854 (9736–11996)	2.47 (2.19–2.76)
World region					
AFR	2756 (2485–3067)	6517 (5951–7120)	8194 (7342–9132)	17468 (15914–19164)	9.46 (8.77–10.2)
AMR	468 (416–524)	604 (555–655)	576 (527–627)	1648 (1513–1782)	0.32 (0.30–0.35)
EMR	1006 (839–1187)	1678 (1371–1998)	2526 (2063–3016)	5210 (4315–6144)	2.53 (2.05–3.08)
EUR	494 (414–590)	252 (223–282)	342 (294–393)	1087 (940–1249)	0.16 (0.14–0.18)
SEAR	2006 (1650–2415)	6721 (5684–7838)	5130 (4295–6059)	13856 (11616–16292)	9.97 (8.48–11.5)
WPR	2047 (1789–2334)	2469 (2208–2760)	3116 (2715–3566)	7632 (6820–8553)	2.90 (2.50–3.39)

Note: Values in parentheses represent equal-tailed 95% credible intervals. Total costs included patient direct medical, direct non-medical, and indirect costs (all undiscounted) in 2020 USD. Costs borne by TB-affected households are categorized as "catastrophic" if they exceed 20% of total household's annual income.

[a] High-TB, high-TB/HIV (HIV-associated TB), and high-MDR/RR-TB (multidrug/rifampicin-resistant TB) burden countries as defined by the World Health Organization.

[b] LIC: Gross national income (GNI) per capita of $1,085 or less; LMIC: GNI per capita of $1,086 to $4,225; UMIC: GNI per capita of $4,256 to $13,205 (World Bank 2021).

Note: All countries include 105 low- and middle-income countries analysed. AFR = African region; AMR = Region of the Americas; EMR = Eastern Mediterranean region; EUR = European region; GDP = gross domestic product; LIC = low-income; LMIC = lower middle-income; SEAR = Southeast Asian region; UMIC = upper middle-income; WPR = Western Pacific region.

Appendix S21. Total costs borne by TB-affected households averted in the base-case (Panel A) and assuming costs for untreated cases are 1.5x treated cases (Panel B) by within-country income quintile comparing infant vaccine to adolescent/adult vaccine.

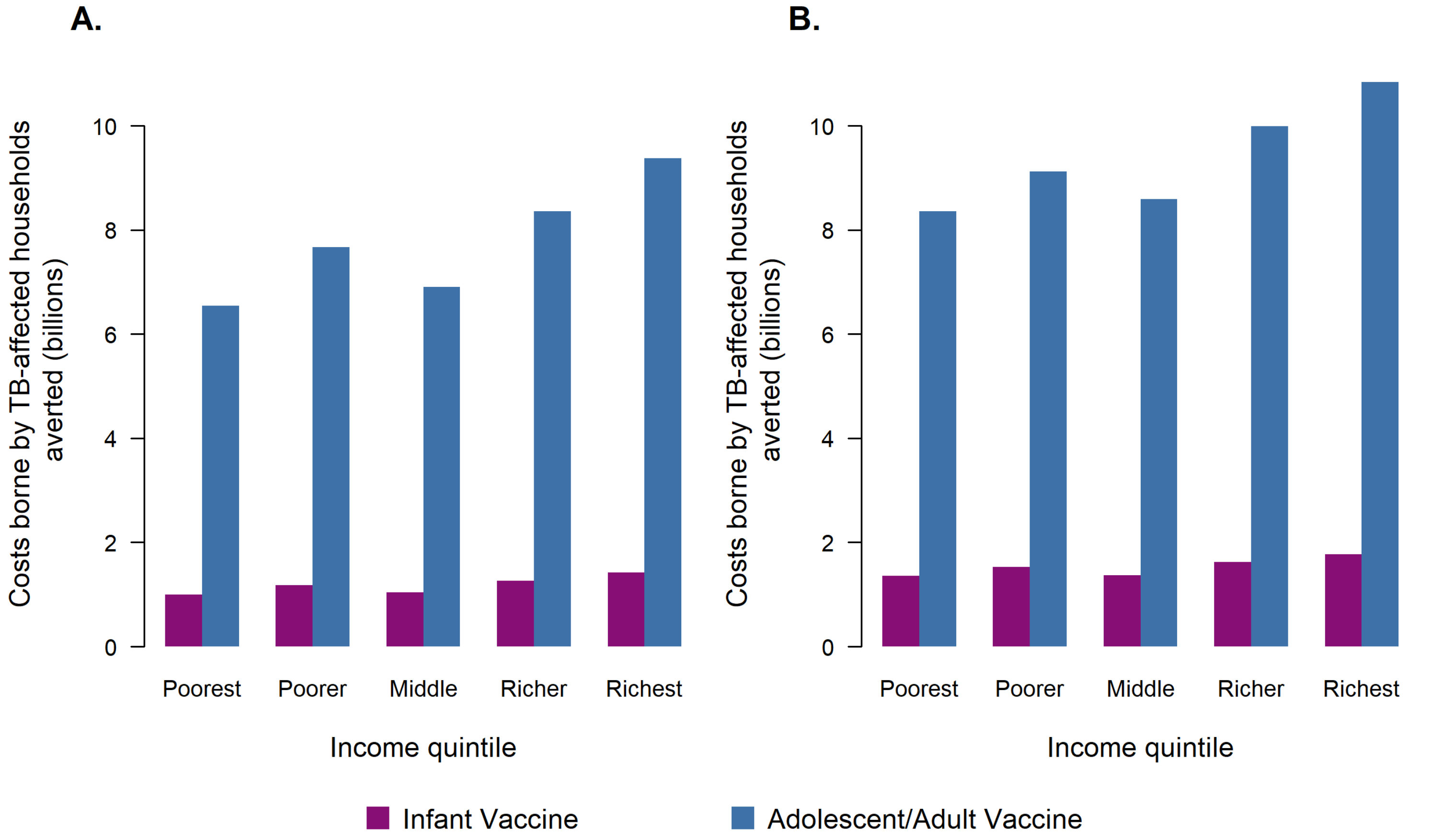

Note: Total costs included patient direct medical, direct non-medical, and indirect costs (all undiscounted) in 2020 USD.

Appendix S22. Number of households with catastrophic costs averted in the base-case (Panel A) and assuming costs for untreated cases are 1.5x treated cases (Panel B) by within-country income quintile comparing infant vaccine to adolescent/adult vaccine.

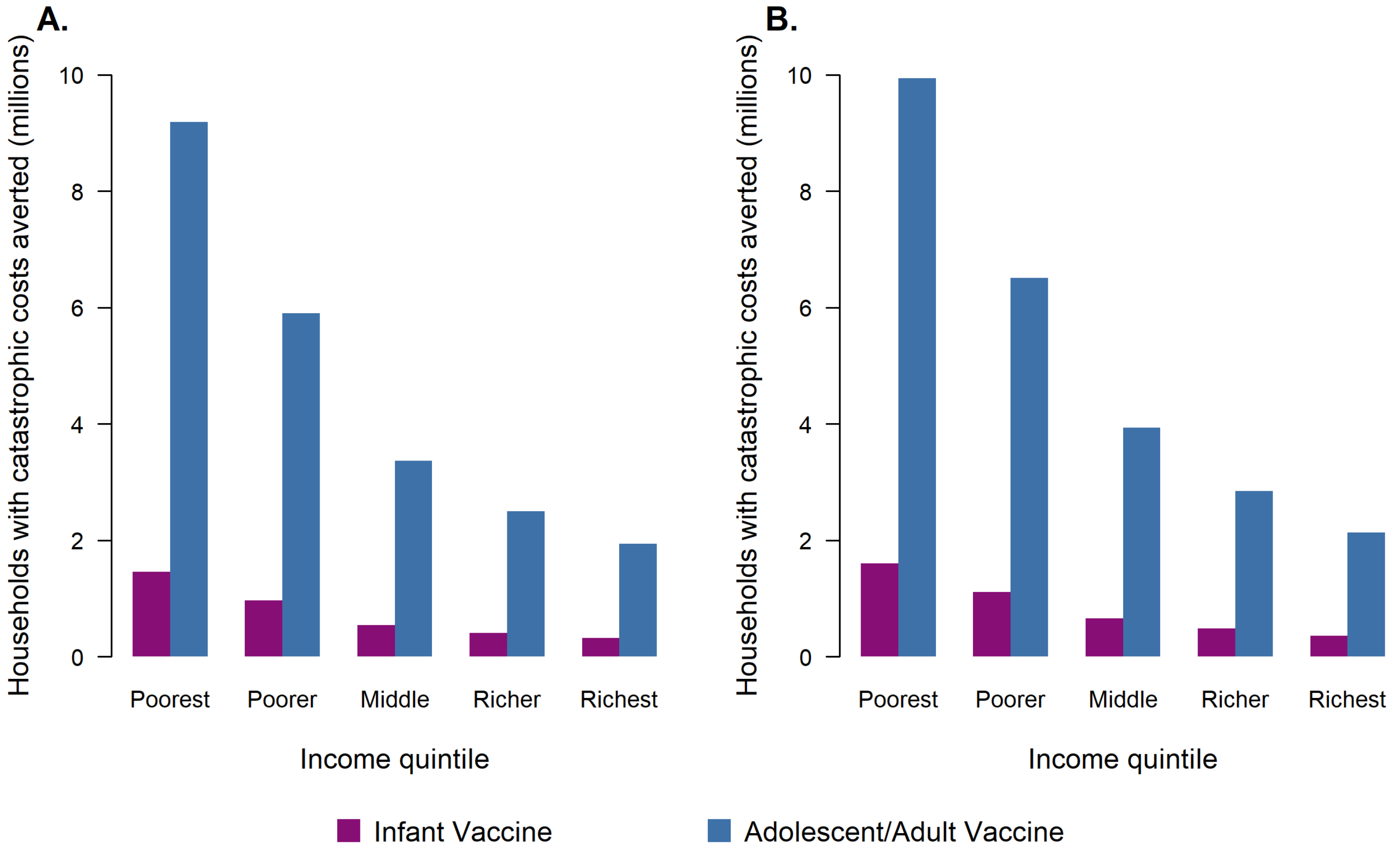

Note: Catastrophic costs included patient direct medical, direct non-medical, and indirect costs. Catastrophic costs are defined as instances where the patient costs incurred during an episode of TB disease exceed 20% of total annual household income.

Appendix S23. Number of households with catastrophic costs averted by infant tuberculosis vaccines (in millions), assuming thresholds of 10%, 20% (base-case), and 25%.

Country grouping	10% threshold		20% threshold		25% threshold	
	Number of households with catastrophic direct medical costs averted	Number of households with catastrophic costs averted	Number of households with catastrophic direct medical costs averted	Number of households with catastrophic costs averted	Number of households with catastrophic direct medical costs averted	Number of households with catastrophic costs averted
All countries	1.15 (1.02–1.28)	5.10 (4.56–5.67)	0.63 (0.56–0.70)	3.69 (3.31–4.08)	0.51 (0.45–0.57)	3.20 (2.87–3.54)
High-TB burden[a]	1.00 (0.87–1.12)	4.64 (4.11–5.21)	0.53 (0.46–0.60)	3.32 (2.94–3.70)	0.43 (0.37–0.48)	2.86 (2.53–3.19)
High-TB/HIV burden[a]	0.85 (0.73–0.97)	3.94 (3.46–4.48)	0.46 (0.39–0.52)	2.84 (2.50–3.21)	0.37 (0.31–0.42)	2.46 (2.17–2.77)
High-MDR/RR-TB burden[a]	0.90 (0.77–1.02)	4.06 (3.54–4.61)	0.48 (0.41–0.55)	2.89 (2.53–3.27)	0.39 (0.33–0.44)	2.49 (2.18–2.81)
Income level[b]						
LIC	0.20 (0.17–0.23)	0.76 (0.65–0.90)	0.12 (0.10–0.14)	0.61 (0.52–0.73)	0.10 (0.08–0.11)	0.55 (0.47–0.66)
LMIC	0.89 (0.76–1.01)	4.03 (3.52–4.60)	0.48 (0.41–0.54)	2.85 (2.50–3.25)	0.38 (0.33–0.44)	2.45 (2.15–2.79)
UMIC	0.07 (0.05–0.08)	0.31 (0.23–0.39)	0.04 (0.03–0.04)	0.23 (0.17–0.29)	0.03 (0.02–0.04)	0.20 (0.15–0.26)
World region						
AFR	0.58 (0.50–0.67)	2.36 (2.05–2.67)	0.32 (0.27–0.37)	1.78 (1.55–2.01)	0.26 (0.22–0.30)	1.56 (1.36–1.77)
AMR	0.006 (0.005–0.007)	0.020 (0.018–0.023)	0.004 (0.003–0.004)	0.015 (0.013–0.016)	0.0030 (0.0026–0.0034)	0.013 (0.011–0.014)
EMR	0.19 (0.15–0.24)	0.66 (0.48–0.86)	0.11 (0.09–0.14)	0.51 (0.38–0.65)	0.09 (0.07–0.12)	0.45 (0.34–0.58)
EUR	0.005 (0.004–0.006)	0.011 (0.009–0.013)	0.003 (0.003–0.004)	0.008 (0.006–0.009)	0.003 (0.002–0.003)	0.007 (0.005–0.008)
SEAR	0.27 (0.21–0.35)	1.50 (1.17–1.91)	0.14 (0.11–0.18)	1.01 (0.80–1.28)	0.11 (0.08–0.14)	0.86 (0.68–1.08)
WPR	0.09 (0.07–0.12)	0.55 (0.41–0.69)	0.05 (0.04–0.06)	0.37 (0.28–0.47)	0.04 (0.03–0.05)	0.32 (0.24–0.40)

Note: Values in parentheses represent equal-tailed 95% credible intervals. Catastrophic costs are defined as instances where the patient costs (either direct medical only or total) incurred during an episode of TB disease exceed either 10%, 20%, or 25% of total annual household income.

[a] High-TB, high-TB/HIV (HIV-associated TB), and high-MDR/RR-TB (multidrug/rifampicin-resistant TB) burden countries as defined by the World Health Organization.

[b] LIC: Gross national income (GNI) per capita of $1,085 or less; LMIC: GNI per capita of $1,086 to $4,225; UMIC: GNI per capita of $4,256 to $13,205 (World Bank 2021).

Note: All countries include 105 low- and middle-income countries analysed. AFR = African region; AMR = Region of the Americas; EMR = Eastern Mediterranean region; EUR = European region; GDP = gross domestic product; LIC = low-income; LMIC = lower middle-income; SEAR = Southeast Asian region; UMIC = upper middle-income; WPR = Western Pacific region.

Appendix S24. Number of households with catastrophic costs averted by adolescent/adult tuberculosis vaccines (in millions), assuming thresholds of 10%, 20% (base-case), and 25%.

Country grouping	10% threshold		20% threshold		25% threshold	
	Number of households with catastrophic direct medical costs averted	Number of households with catastrophic costs averted	Number of households with catastrophic direct medical costs averted	Number of households with catastrophic costs averted	Number of households with catastrophic direct medical costs averted	Number of households with catastrophic costs averted
All countries	6.97 (6.43–7.52)	32.3 (30.0–34.6)	3.75 (3.46–4.05)	22.9 (21.4–24.5)	3.01 (2.78–3.25)	19.7 (18.4–21.1)
High-TB burden[a]	5.91 (5.40–6.43)	28.9 (26.7–31.2)	3.12 (2.85–3.40)	20.2 (18.7–21.8)	2.48 (2.27–2.71)	17.4 (16.0–18.7)
High-TB/HIV burden[a]	4.89 (4.43–5.36)	24.4 (22.3–26.7)	2.57 (2.32–2.84)	17.3 (15.8–18.8)	2.05 (1.84–2.26)	14.8 (13.6–16.1)
High-MDR/RR-TB burden[a]	5.41 (4.91–5.93)	25.7 (23.4–28.0)	2.87 (2.60–3.15)	17.9 (16.4–19.4)	2.29 (2.07–2.52)	15.3 (14.0–16.6)
Income level[b]						
LIC	1.04 (0.95–1.15)	4.20 (3.83–4.59)	0.60 (0.54–0.66)	3.31 (3.02–3.62)	0.49 (0.44–0.55)	2.97 (2.70–3.25)
LMIC	5.12 (4.62–5.62)	24.9 (22.6–27.1)	2.71 (2.44–2.99)	17.3 (15.8–18.8)	2.16 (1.95–2.38)	14.8 (13.6–16.1)
UMIC	0.80 (0.72–0.88)	3.21 (2.90–3.53)	0.44 (0.39–0.48)	2.25 (2.01–2.50)	0.35 (0.32–0.39)	1.94 (1.73–2.17)
World region						
AFR	2.62 (2.41–2.86)	11.3 (10.5–12.1)	1.44 (1.32–1.58)	8.52 (7.93–9.16)	1.17 (1.06–1.28)	7.49 (6.96–8.06)
AMR	0.12 (0.11–0.13)	0.41 (0.38–0.44)	0.07 (0.06–0.07)	0.30 (0.28–0.32)	0.06 (0.05–0.06)	0.26 (0.24–0.28)
EMR	0.87 (0.73–1.03)	3.11 (2.52–3.77)	0.51 (0.42–0.60)	2.35 (1.92–2.83)	0.42 (0.35–0.49)	2.08 (1.70–2.48)
EUR	0.10 (0.09–0.11)	0.22 (0.20–0.24)	0.06 (0.05–0.07)	0.15 (0.13–0.17)	0.05 (0.04–0.06)	0.13 (0.11–0.15)
SEAR	2.40 (2.02–2.80)	13.3 (11.4–15.3)	1.22 (1.02–1.42)	8.94 (7.68–10.3)	0.96 (0.80–1.12)	7.54 (6.48–8.64)
WPR	0.85 (0.75–0.96)	3.92 (3.42–4.52)	0.45 (0.40–0.51)	2.63 (2.29–3.04)	0.36 (0.32–0.41)	2.23 (1.93–2.57)

Note: Values in parentheses represent equal-tailed 95% credible intervals. Catastrophic costs are defined as instances where the patient costs (either direct medical only or total) incurred during an episode of TB disease exceed either 10%, 20%, or 25% of total annual household income.

[a] High-TB, high-TB/HIV (HIV-associated TB), and high-MDR/RR-TB (multidrug/rifampicin-resistant TB) burden countries as defined by the World Health Organization.

[b] LIC: Gross national income (GNI) per capita of $1,085 or less; LMIC: GNI per capita of $1,086 to $4,225; UMIC: GNI per capita of $4,256 to $13,205 (World Bank 2021).

Note: All countries include 105 low- and middle-income countries analysed. AFR = African region; AMR = Region of the Americas; EMR = Eastern Mediterranean region; EUR = European region; GDP = gross domestic product; LIC = low-income; LMIC = lower middle-income; SEAR = Southeast Asian region; UMIC = upper middle-income; WPR = Western Pacific region.

Appendix S25. Partial rank correlation coefficients for the number of cases of catastrophic costs projected to be averted by the adolescent/adult TB vaccine.

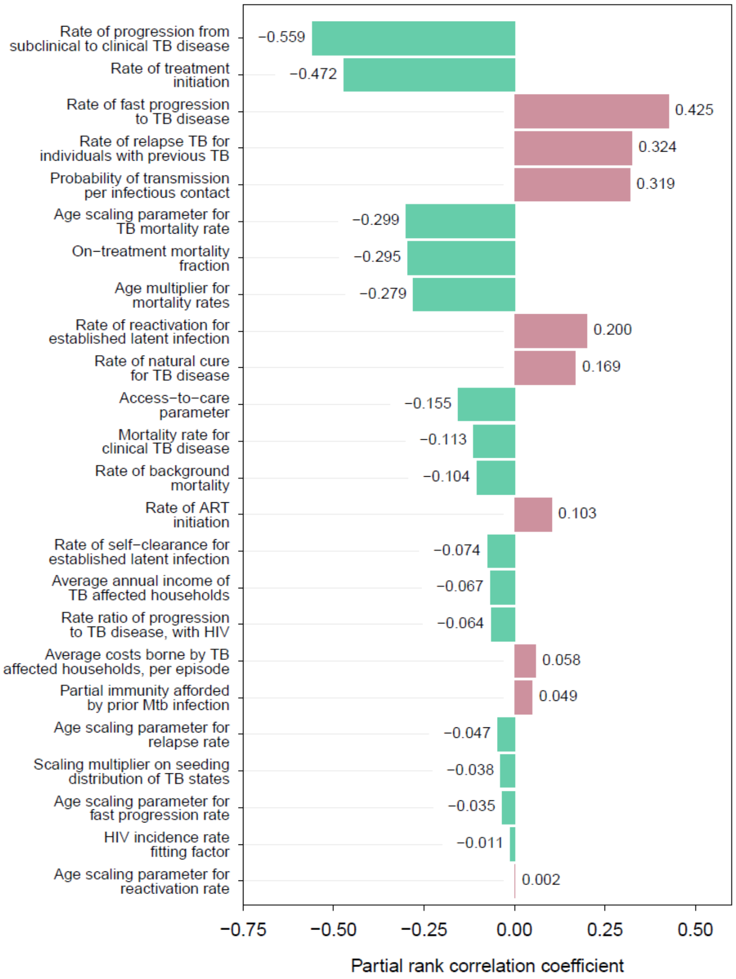

Note: TB = tuberculosis. HIV = Human Immunodeficiency Virus. ART = antiretroviral therapy for HIV. Negative (positive) values indicate a negative (positive) relationship with the outcome. Greater magnitude indicates a stronger relationship. Values in figure represent the median PRCC for each parameter across all individual country models.

Appendix S26. Partial rank correlation coefficients for the number of cases of catastrophic costs projected to be averted by the infant TB vaccine.

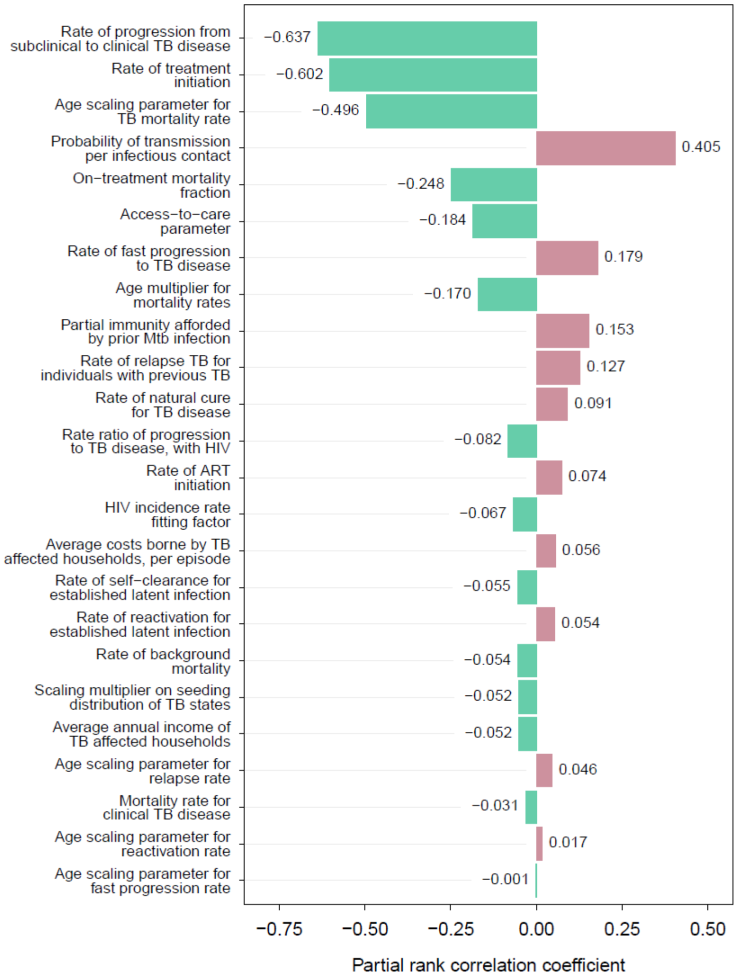

Note: TB = tuberculosis. HIV = Human Immunodeficiency Virus. ART = antiretroviral therapy for HIV. Negative (positive) values indicate a negative (positive) relationship with the outcome. Greater magnitude indicates a stronger relationship. Values in figure represent the median PRCC for each parameter across all individual country models.

References

1. Clark RA, Mukandavire C, Portnoy A, Weerasuriya CK, Deol AK, Scarponi D, et al. The impact of alternative delivery strategies for novel tuberculosis vaccines in low- and middle-income countries: a modelling study. Lancet Glob Health. 2023;11(4):e546-e55.

2. Frascella B, Richards AS, Sossen B, Emery JC, Odone A, Law I, et al. Subclinical Tuberculosis Disease-A Review and Analysis of Prevalence Surveys to Inform Definitions, Burden, Associations, and Screening Methodology. Clin Infect Dis. 2021;73(3):e830-e41.

3. Emery JC, Richards AS, Dale KD, McQuaid CF, White RG, Denholm JT, et al. Self-clearance of Mycobacterium tuberculosis infection: implications for lifetime risk and population at-risk of tuberculosis disease. Proc Biol Sci. 2021;288(1943):20201635. https://doi.org/10.1098/rspb.2020.1635

4. World Health Organization. Guidelines for treatment of drug-susceptible tuberculosis and patient care: 2017 update. Last updated: 24 April 2017. Available at: https://apps.who.int/iris/handle/10665/255052

5. World Health Organization. Treatment of drug-susceptible tuberculosis: rapid communication. Last updated: 11 June 2021. Available at: https://apps.who.int/iris/handle/10665/341729

6. Kwan CK, Ernst JD. HIV and tuberculosis: a deadly human syndemic. Clin Microbiol Rev. 2011;24(2):351-76.

7. Vynnycky E, Fine PE. The natural history of tuberculosis: the implications of age-dependent risks of disease and the role of reinfection. Epidemiol Infect. 1997;119(2):183-201.

8. Suthar AB, Lawn SD, del Amo J, Getahun H, Dye C, Sculier D, et al. Antiretroviral therapy for prevention of tuberculosis in adults with HIV: a systematic review and meta-analysis. PLoS Med. 2012;9(7):e1001270.

9. Harling G, Ehrlich R, Myer L. The social epidemiology of tuberculosis in South Africa: a multilevel analysis. Soc Sci Med. 2008;66(2):492-505. https://doi.org/10.1016/j.socscimed.2007.08.026

10. Hoa NB, Tiemersma EW, Sy DN, Nhung NV, Gebhard A, Borgdorff MW, et al. Household expenditure and tuberculosis prevalence in VietNam: prediction by a set of household indicators. Int J Tuberc Lung Dis. 2011;15(1):32-7.

11. Hossain S, Quaiyum MA, Zaman K, Banu S, Husain MA, Islam MA, et al. Socio economic position in TB prevalence and access to services: results from a population prevalence survey and a facility-based survey in Bangladesh. PLoS One. 2012;7(9):e44980. https://doi.org/10.1371/journal.pone.0044980

12. Kapata N, Chanda-Kapata P, Ngosa W, Metitiri M, Klinkenberg E, Kalisvaart N, et al. The Prevalence of Tuberculosis in Zambia: Results from the First National TB

Prevalence Survey, 2013-2014. PLoS One. 2016;11(1):e0146392. https://doi.org/10.1371/journal.pone.0146392

13. Migambi P, Gasana M, Uwizeye CB, Kamanzi E, Ndahindwa V, Kalisvaart N, et al. Prevalence of tuberculosis in Rwanda: Results of the first nationwide survey in 2012 yielded important lessons for TB control. PLoS One. 2020;15(4):e0231372. https://doi.org/10.1371/journal.pone.0231372

14. Oxlade O, Murray M. Tuberculosis and poverty: why are the poor at greater risk in India? PLoS One. 2012;7(11):e47533. https://doi.org/10.1371/journal.pone.0047533

15. Singh SK, Kashyap GC, Puri P. Potential effect of household environment on prevalence of tuberculosis in India: evidence from the recent round of a cross-sectional survey. BMC Pulm Med. 2018;18(1):66. https://doi.org/10.1186/s12890-018-0627-3

16. Siroka A, Law I, Macinko J, Floyd K, Banda RP, Hoa NB, et al. The effect of household poverty on tuberculosis. Int J Tuberc Lung Dis. 2016;20(12):1603-8. https://doi.org/10.5588/ijtld.16.0386

17. van Leth F, Guilatco RS, Hossain S, Van't Hoog AH, Hoa NB, van der Werf MJ, et al. Measuring socio-economic data in tuberculosis prevalence surveys. Int J Tuberc Lung Dis. 2011;15 Suppl 2:58-63. https://doi.org/10.5588/ijtld.10.0417

18. Yates TA, Ayles H, Leacy FP, Schaap A, Boccia D, Beyers N, et al. Socio-economic gradients in prevalent tuberculosis in Zambia and the Western Cape of South Africa. Trop Med Int Health. 2018;23(4):375-90. https://doi.org/10.1111/tmi.13038

19. Foster N, Nguyen HV, Nguyen NV, Nguyen HB, Tiemersma EW, Cobelens FGJ, et al. Social determinants of the changing tuberculosis prevalence in Việt Nam: Analysis of population-level cross-sectional studies. PLoS Med. 2022;19(3):e1003935. https://doi.org/10.1371/journal.pmed.1003935

20. Iskauskas A. hmer: History Matching and Emulation Package. R package. 17 May 2022. Available at: https://CRAN.R-project.org/package=hmer (accessed 12 July 2022).

21. Scarponi D, Iskauskas A, Clark RA, Vernon I, McKinley TJ, Goldstein M, et al. Demonstrating Multi-Country Calibration of a Tuberculosis Model Using New History Matching and Emulation Package - Hmer. medRxiv; 2022. doi: https://doi.org/10.1101/2022.05.13.22275052.

22. Jabot F, Faure T, Dumoulin N, Albert C. EasyABC: Efficient Approximate Bayesian Computation Sampling Schemes. R package. 2015. Available at: https://CRAN.R-project.org/package=EasyABC (accessed April 2022).

23. World Health Organization. WHO Preferred Product Characteristics for New Tuberculosis Vaccines. Geneva: World Health Organization. Licence: CC BY-NC-SA 3.0 IGO. 13 July 2018. https://www.who.int/publications/i/item/WHO-IVB-18.06 (accessed 18 October 2021).

24. Abubakar I, Pimpin L, Ariti C, Beynon R, Mangtani P, Sterne JA, et al. Systematic review and meta-analysis of the current evidence on the duration of protection by bacillus Calmette-Guérin vaccination against tuberculosis. Health Technol Assess. 2013;17(37):1-372, v-vi.

25. Blackwood JC, Cummings DA, Broutin H, Iamsirithaworn S, Rohani P. Deciphering the impacts of vaccination and immunity on pertussis epidemiology in Thailand. Proc Natl Acad Sci U S A. 2013;110(23):9595-600.

26. Lewnard JA, Grad YH. Vaccine waning and mumps re-emergence in the United States. Sci Transl Med. 2018;10(433).

27. Gavi The Vaccine Alliance. Country hub. Geneva: Gavi, The Vaccine Alliance. https://www.gavi.org/programmes-impact/country-hub (accessed 15 April 2022).

28. UNICEF. Vaccination and Immunization Statistics. Last updated: July 2021. Available at: https://data.unicef.org/topic/child-health/immunization/

29. Harris RC, Sumner T, Knight GM, Zhang H, White RG. Potential impact of tuberculosis vaccines in China, South Africa, and India. Sci Transl Med. 2020;12(564). https://doi.org/10.1126/scitranslmed.aax4607

30. United Nations, Department of Economic and Social Affairs, Population Division. World Population Projections, 2019 Revision. Last updated: 28 August 2019. Available at: https://population.un.org/wpp/Download/Standard/Population/

31. Tiemersma EW, van der Werf MJ, Borgdorff MW, Williams BG, Nagelkerke NJ. Natural history of tuberculosis: duration and fatality of untreated pulmonary tuberculosis in HIV negative patients: a systematic review. PLoS One. 2011;6(4):e17601. https://doi.org/10.1371/journal.pone.0017601

32. Quaife M, Houben R, Allwood B, Cohen T, Coussens AK, Harries AD, et al. Post-tuberculosis mortality and morbidity: valuing the hidden epidemic. Lancet Respir Med. 2020;8(4):332-3. https://doi.org/10.1016/S2213-2600(20)30039-4

33. World Health Organization. WHO TB incidence estimates disaggregated by age group, sex and risk factor. Last updated: December 2021. Available at: https://www.who.int/teams/global-tuberculosis-programme/data

34. World Health Organization. Case Notifications. Last updated: December 2021. Available at: https://www.who.int/teams/global-tuberculosis-programme/data

35. Emery J. Subclinical TB disease: lessons and questions from field to lab. Presentation at TB Science, 2020.

36. Abu-Raddad LJ, Sabatelli L, Achterberg JT, Sugimoto JD, Longini IM, Jr., Dye C, et al. Epidemiological benefits of more-effective tuberculosis vaccines, drugs, and diagnostics. Proc Natl Acad Sci USA. 2009;106(33):13980-5. https://doi.org/10.1073/pnas.0901720106

37. Dye C, Williams BG. Eliminating human tuberculosis in the twenty-first century. J R Soc Interface. 2008;5(23):653-62. https://doi.org/10.1098/rsif.2007.1138

38. Marx FM, Dunbar R, Enarson DA, Williams BG, Warren RM, van der Spuy GD, et al. The temporal dynamics of relapse and reinfection tuberculosis after successful treatment: a retrospective cohort study. Clin Infect Dis. 2014;58(12):1676-83. https://doi.org/10.1093/cid/ciu186

39. Gomes GM, Franco AO, Gomes MC, Medley GF. The reinfection threshold promotes variability in tuberculosis epidemiology and vaccine efficacy. Proc Biol Sci. 2004;271(1539):617-23. https://doi.org/10.1098/rspb.2003.2606

40. Dangisso MH, Woldesemayat EM, Datiko DG, Lindtjørn B. Long-term outcome of smear-positive tuberculosis patients after initiation and completion of treatment: A ten-year retrospective cohort study. PLoS One. 2018;13(3):e0193396. https://doi.org/10.1371/journal.pone.0193396

41. World Health Organization. Treatment Outcomes. Last updated: December 2021. Available at: https://www.who.int/teams/global-tuberculosis-programme/data

42. Sutherland I, Svandová E, Radhakrishna S. The development of clinical tuberculosis following infection with tubercle bacilli. 1. A theoretical model for the development of clinical tuberculosis following infection, linking from data on the risk of tuberculous infection and the incidence of clinical tuberculosis in the Netherlands. Tubercle. 1982;63(4):255-68. https://doi.org/10.1016/s0041-3879(82)80013-5

43. Gomes GM, Rodrigues P, Hilker FM, Mantilla-Beniers NB, Muehlen M, Cristina Paulo A, et al. Implications of partial immunity on the prospects for tuberculosis control by post-exposure interventions. J Theor Biol. 2007;248(4):608-17. https://doi.org/10.1016/j.jtbi.2007.06.005

44. Mberi MN, Kuonza LR, Dube NM, Nattey C, Manda S, Summers R. Determinants of loss to follow-up in patients on antiretroviral treatment, South Africa, 2004-2012: a cohort study. BMC Health Serv Res. 2015;15:259. https://doi.org/10.1186/s12913-015-0912-2

45. Van Cutsem G, Ford N, Hildebrand K, Goemaere E, Mathee S, Abrahams M, et al. Correcting for mortality among patients lost to follow up on antiretroviral therapy in South Africa: a cohort analysis. PLoS One. 2011;6(2):e14684. https://doi.org/10.1371/journal.pone.0014684

46. Johansson KA, Robberstad B, Norheim OF. Further benefits by early start of HIV treatment in low income countries: survival estimates of early versus deferred antiretroviral therapy. AIDS Res Ther. 2010;7(1):3. https://doi.org/10.1186/1742-6405-7-3

47. Fatti G, Mothibi E, Meintjes G, Grimwood A. Antiretroviral treatment outcomes amongst older adults in a large multicentre cohort in South Africa. PLoS One. 2014;9(6):e100273. https://doi.org/10.1371/journal.pone.0100273

48. Vazquez F. A systematic review and meta-analysis of the effect of HIV status on the incidence of tuberculosis disease among individuals with latent Mycobacterium tuberculosis infection. [Masters Dissertation]. 2019.

49. Ackah AN, Coulibaly D, Digbeu H, Diallo K, Vetter KM, Coulibaly IM, et al. Response to treatment, mortality, and CD4 lymphocyte counts in HIV-infected persons with tuberculosis in Abidjan, Côte d'Ivoire. Lancet. 1995;345(8950):607-10. https://doi.org/10.1016/s0140-6736(95)90519-7

50. Mukadi YD, Maher D, Harries A. Tuberculosis case fatality rates in high HIV prevalence populations in sub-Saharan Africa. Aids. 2001;15(2):143-52. https://doi.org/10.1097/00002030-200101260-00002

51. World Health Organization. WHO releases new global lists of high-burden countries for TB, HIV-associated TB and drug-resistant TB. 17 June 2021. https://www.who.int/news/item/17-06-2021-who-releases-new-global-lists-of-high-burden-countries-for-tb-hiv-associated-tb-and-drug-resistant-tb (accessed 1 July 2021).

52. Portnoy A, Yamanaka T, Nguhiu P, Nishikiori N, Floyd K, Baena IG, et al. Costs incurred by people receiving TB treatment in low- and middle-income countries: a meta-regression analysis.

53. Vassall A, Sweeney S, Kahn J, Gomez GB, Bollinger L, Marseille E, et al. Global Health Costing Consortium reference case for estimating the costs of global health services and interventions. 2017. https://ghcosting.org/pages/standards/reference_case. (accessed 29 July 2022).
